# Molecular evidences confirm the taxonomic separation of two sympatric congeneric species (Mollusca, Gastropoda, Neritidae, *Neritina*)

**DOI:** 10.3897/zookeys.904.46790

**Published:** 2020-01-16

**Authors:** Cristiane Xerez Barroso, João Eduardo Pereira de Freitas, Helena Matthews-Cascon, Luis Ernesto Arruda Bezerra, Tito Monteiro da Cruz Lotufo

**Affiliations:** 1 Graduate Program on Marine Tropical Sciences, Instituto de Ciências do Mar -LABOMAR, Universidade Federal do Ceará, Av. da Abolição, 3207, Meireles, CEP: 60165-081, Fortaleza, CE, Brazil; 2 Laboratório de Invertebrados Marinhos do Ceará - LIMCE, Departamento de Biologia, Centro de Ciências, Universidade Federal do Ceará, Rua Campus do Pici, s/n, Bloco 909, Pici, CEP: 60440-900, Fortaleza, CE, Brazil; 3 Marine Vertebrate Evolution and Conservation Lab - EvolVe, Departamento de Biologia, Centro de Ciências, Universidade Federal do Ceará, Rua Campus do Pici, s/n, Bloco 909, Pici, CEP: 60440-900, Fortaleza, CE, Brazil; 4 Laboratório de Zoobentos, Instituto de Ciências do Mar - LABOMAR, Universidade Federal do Ceará, Av. da Abolição, 3207, Meireles, CEP: 60165-081, Fortaleza, CE, Brazil; 5 Laboratório de Biologia Recifal - BIOREC, Instituto Oceanográfico, Universidade de São Paulo, Praça do Oceanográfico, 191, CEP: 05508-120, São Paulo, SP, Brazil

**Keywords:** Brazilian Province, Caribbean Province, geographic distribution, neritids, species delimitation

## Abstract

A reliable taxonomy, together with more accurate knowledge of the geographical distribution of species, is a fundamental element for the study of biodiversity. Multiple studies on the gastropod family Neritidae record three species of the genus *Neritina* in the Brazilian Province: *Neritina zebra* (Bruguière, 1792), *Neritina virginea* (Linnaeus, 1758), and *Neritina meleagris* Lamarck, 1822. While *N. zebra* has a well-established taxonomic status and geographical distribution, the same cannot be said regarding its congeners. A widely cited reference for the group in Brazil considers *N. meleagris* a junior synonym of *N. virginea*. Using a molecular approach (phylogenetic, species delimitation, and statistical parsimony network analyses), based on two mitochondrial markers (COI and 16S), this study investigated if *N. virginea* and *N. meleagris* are distinct species. The molecular results confirmed the existence of two strongly supported distinct taxonomic entities in the Brazilian Province, which is consistent with the morphological descriptions previously proposed for *N. virginea* and *N. meleagris*. These species occur in sympatry in the intertidal sandstone formations of Northeastern Brazil. Despite the great variation in the colour patterns of the shells, the present study reinforced previous observations that allowed the differentiation of these two species based on these patterns. It also emphasized the importance of the separation of these two clades in future studies, especially those conducted in the Brazilian Province, since these species may cohabit.

## Introduction

Molluscs from the gastropod family Neritidae are the most diverse members of Neritimorpha (Kano et al. 2002), with some groups within this family having variable shell colouration patterns (e.g., Russell 1941; Tan and Clements 2008; Eichhorst 2016). Due to the great variety of colour patterns, the delimitation of different species could be hampered, especially if they are closely related and live in sympatry (e.g., Huang 1997; Blanco et al. 2014). This may explain the disparate estimates in the literature of the number of species of *Neritina* reported for the Brazilian Province.

Several studies report three species of the genus *Neritina* on the Brazilian coast: *Neritina zebra* (Bruguière, 1792), *Neritina virginea* (Linnaeus, 1758), and *Neritina meleagris* Lamarck, 1822 (e.g., Baker 1923; Russell 1941; Rios 1975; Matthews-Cascon et al. 1990; Díaz and Puyana 1994; Quintero-Galvis and Castro 2013; Eichhorst 2016). While *N. zebra* has a well-established taxonomic status and geographical distribution (Matthews-Cascon et al. 1990; Rios 2009; Barroso et al. 2012; Eichhorst 2016), there is uncertainty regarding its congeners. The shell catalogues of Rios (1985, 1994, 2009), a widely cited reference in studies conducted in Brazil, state that only two species occur in the Brazilian Province: *N. virginea* and *N. zebra*. In these compendia, *N. meleagris* is considered a junior synonym of *N. virginea* without any justification. Quintero-Galvis and Castro (2013), using a molecular phylogenetic approach to analyse specimens from the Colombian coast (Caribbean Province), concluded that *N. meleagris* and *N. virginea* are phylogenetically close, but different species. Since these species have a wide geographic distribution, encompassing the Caribbean and Brazilian Provinces (Barroso et al. 2016; Eichhorst 2016), that are separated by a recognized biogeographic barrier (the Amazon-Orinoco outflow) (Floeter et al. 2008; Barroso et al. 2016), the inclusion of specimens from both biogeographical provinces in phylogenetic analyses is desirable.

Since a reliable taxonomy, together with a more accurate knowledge about the geographical distribution of species, is fundamental to the study of biodiversity (Wheeler et al. 2004), the present study aims to investigate if *N. virginea* and *N. meleagris* are two distinct species, using molecular data (phylogenetic, species delimitation, and statistical parsimony network analyses).

## Methods

We collected specimens from Barra Grande beach (Piauí State) (2°54.125'S, 41°24.573'W) and Camocim beach (Ceará State) (02°51.778'S, 41°51.57'W), both located in Northeastern Brazil, and preserved them in 95% ethanol. We identified the species using the literature (Russell 1941; Matthews-Cascon et al. 1990; Eichhorst 2016), primarily based on the shape and colour patterns of the shells. Specimens were collected under SISBIO permit no. 57473-3 and deposited in the malacological collection “Prof. Henry Ramos Matthews” – series B (CMPHRM-B) of Universidade Federal do Ceará (UFC). A total of 17 specimens, eight newly sequenced and nine already published by Quintero-Galvis and Castro (2013) and available on GenBank, were used for phylogenetic reconstruction (Table 1). All sequences used were attributed to nominal species considered valid in the literature (see Aktipis and Giribet 2010; Cook et al. 2010; Page et al. 2013; Quintero-Galvis and Castro 2013).

**Table 1.** List of species included in the phylogenetic, species delimitation, and statistical parsimony network analyses. The voucher number of species collected in NE Brazil and the accession numbers of the sequences obtained in the present study and from GenBank are indicated. The numbers in parentheses next to the GenBank accession number correspond to each of the specimens analysed in the present study (see Figs 1, 3).

Family/Species	Locality	Voucher No.	Accession No.		References^b^
			COI	16S^a^	
**Outgroups**					
** Phenacolepadidae **					
*Thalassonerita naticoidea* (A. H. Clarke, 1989)	Gulf of Mexico	–	FJ977768	FJ977721	1
** Neritidae **					
*Nerita fulgurans* Gmelin, 1791	Colombia	–	JX646664	JX646655	2
*Nerita tessellata* Gmelin, 1791	Colombia	–	JX646663	JX646654	2
*Nerita peloronta* Linnaeus, 1758	Colombia	–	JX646665	JX646656	2
*Nerita versicolor* Gmelin, 1791	Colombia	–	JX646666	JX646658	2
*Neritina piratica* Russell, 1940	Colombia	–	JX646669	JX646660	2
*Neritina usnea* (Röding, 1798)	Colombia	–	JX646670	JX646661	2
*Neritina punctulata* Lamarck, 1816	Colombia	–	JX646667	JX646657	2
**Ingroup**					
*Neritina meleagris* Lamarck, 1822	Colombia	–	JX646671	JX646662	2
	Camocim, Ceará, Brazil	CMPHRM 6408B	MK628548 (1)	MK628556 (1)	3
	Barra Grande, Piauí, Brazil	CMPHRM 6409B	MK628549 (2), MK628550, (3) MK628551 (4)	MK628557 (2), MK628558 (3), MK628559 (4)	3
*Neritina virginea* (Linnaeus, 1758)	Colombia	–	JX646668	JX646659	2
	Panama	–	JF810998 to JF811004^c^ and FJ977766	–	1, 4
	Puerto Rico	–	FJ348932 to FJ348975	–	5
	Camocim, Ceará, Brazil	CMPHRM 6410B	MK628552 (1)	MK628560 (1)	3
	Barra Grande, Piauí, Brazil	CMPHRM 6411B	MK628553 (2), MK628554 (3), MK628555 (4)	MK628561 (2), MK628562 (3), MK628563 (4)	3

^a^Our 16S sequences deposited in GenBank are longer than those used for the construction of the molecular phylogenetic hypothesis. ^b^1. Aktipis and Giribet (2010); 2. Quintero-Galvis and Castro (2013); 3. present study; 4. Page et al. (2013); and 5. Cook et al. (2010). ^c^For the statistical parsimony network analysis, the haplotypes JF811001 and JF811002 had frequency 2 (Page et al. 2013).

We extracted whole genomic DNA from the foot muscle of specimens, using the Qiagen DNeasy Blood & Tissue Kit (Qiagen, Valencia, CA, USA). The quality and integrity of the DNA obtained were evaluated in a micro-volume spectrophotometer. Amplification of double-stranded fragments from the cytochrome c oxidase I (COI) and 16S mitochondrial genes was achieved by polymerase chain reaction (PCR) using newly developed neritid-specific custom primers for the 16S gene [(16SNer_F 5’ACTACTCCGCCTGTTTATCAAA3’) and (16SNer_R 5’GGGCTTAAACCTAATGCACTT3’)] and modified versions of Folmer et al. (1994) primers for the COI gene [(LCO1490_mod 5’ATTCTACGAATCAYAAAGAYATTGG3’) and (HCO2198_mod 5’TAWACTTCAGGATGACCRAAAAATCA3’)]. The PCR was carried out using GoTaq Green Master Mix (Promega Corporation), 1.25 μL of each primer (10 μM stock), and 100 ng of DNA template in a 25 μL reaction volume. The PCR cycles for COI and 16S amplification consisted of an initial denaturation step at 95 °C for 2 min, followed by 35 cycles of denaturation at 95 °C for 30 s, annealing at 48–49 °C for 45 s and extension at 72 °C for 1 min, and a final extension at 72 °C for 5 min. The PCR products were then examined using gel electrophoresis on 1.3% Tris-Borate-EDTA-agarose gel stained with SYBR Safe DNA Gel Stain (Invitrogen). The PCR products showing strong bands in gel electrophoresis were purified with IllustraExoProStar - 1 Step (GE Healthcare Life Sciences), following its standard protocol, and sent for Sanger sequencing (Macrogen Inc., South Korea).

The forward and reverse sequences for each gene fragment were edited using Geneious v. 7.1 (Biomatters). The concatenated alignments of COI and 16S were conducted using the MAFFT program with the G-INS-I algorithm (Katoh and Standley 2013) using the default parameters, with additional inspection by eye for accuracy (see Suppl. material 1). As our 16S sequences (650 bp for *N. virginea* and 651 bp for *N. meleagris*) were longer than those available on GenBank, we used a minor homologous region in the alignment of this gene. However, we deposited the full 650–651 bp 16S sequences in GenBank. The combined dataset contained 1124 bp (639 bp for COI and 485 bp for 16S). Evolutionary relationships were estimated for the concatenated genetic markers using Bayesian inference (BI) and maximum likelihood (ML) analyses. The best-fit evolution models were determined using PartitionFinder (Lanfear et al. 2012), considering the positions of the codon for the COI gene, which codes for protein, and a single partition for the 16S gene. The corrected Bayesian Information Criterion (BIC) was used to select among the options. PartitionFinder selected respectively GTR + I, F81, and HKY + G as the best model for the three positions of the codon in COI, and GTR + G for 16S. Bayesian inference, using the previously mentioned partitions and models, was performed using the MrBayes program (Ronquist et al. 2012) and the dataset was run for 3 × 10^7^ generations, with Markov chains sampled every 1000 generations, and the standard 25% burn-in calculated. Convergence was checked using Tracer 1.6 (http://beast.bio.ed.ac.uk/Tracer). Tree branches were considered strongly supported if posterior probabilities were ≥ 0.90. Randomized accelerated maximum likelihood (RAxML) (Stamatakis 2006) was used to generate a ML tree with partitions under the evolution model GTR + G and with 1 × 10^4^ replications. Branches with bootstrap values greater than 70 were considered strongly supported. Phylogenetic trees were drawn and edited in FigTree 1.4.3 (http://tree.bio.ed.ac.uk/software/figtree/).

For the species delimitation analyses, we initially constructed a distance matrix based on the Kimura 2-parameter (K2P) model, using the COI sequences, in the MEGA 6.0.6 software (Tamura et al. 2013). This matrix was analysed with the default settings of the Automatic Barcode Gap Discovery method (ABGD) (Puillandre et al. 2012) (available at http://www.abi.snv.jussieu.fr/public/abgd/abgdweb.html). We also used the Species Delimitation plugin v1.04 for Geneious v. 7.1 (Masters et al. 2011) with two data sets: (1) the results of our Bayesian concatenated phylogenetic analysis (COI + 16S), and (2) the results of a neighbor-joining tree based on the K2P model with 10,000 bootstrap replicates using COI sequences generated in MEGA 6.0.6. In this analysis, we calculated (1) the mean distance between the members within the clade (Intra Dist), (2) the mean distance of those individuals to the nearest clade (Inter Dist-closest), (3) the ratio between Intra Dist and Inter Dist-closest, and (4) the P ID, which represents the mean probability (95% confidence interval) of correctly identifying an unknown member of the putative species to fit inside (Strict P ID), or at least to be the sister group of (Liberal P ID), the species clade in a tree (Masters et al. 2011).

A statistical parsimony network analysis was conducted with COI sequences (347 bp), using the TCS algorithm (Clement et al. 2002) implemented in PopART v. 1.7.2 (Leigh and Bryant 2015). The sequence alignment step followed the same procedures already described in our phylogenetic analysis protocol (see Suppl. material 1). In addition to the *N. virginea* and *N. meleagris* sequences generated in this study, we included 55 sequences of *N. virginea* from island (Puerto Rico: 44 sequences) and continental (Panama: 10; Colombia: 1) locations in the Caribbean Province (Aktipis and Giribet 2010; Cook et al. 2010; Page et al. 2013; Quintero-Galvis and Castro 2013) (Table 1). Along with phylogenetic and species delimitation analyses, we also included the only COI sequence available on GenBank attributed to *N. meleagris* from Colombia (Quintero-Galvis and Castro 2013) (Table 1).

The shells and opercula of the specimens of *N. virginea* and *N. meleagris* submitted to molecular analyses were observed and photographed under a stereomicroscope. A scanning electron microscope (SEM) was used to view their radulae (two females of *N. virginea* and two males and one female of *N. meleagris*) in the Analytical Facility of UFC (Central Analítica, UFC). This information was collected in order to compare our results with the information available in the literature.

## Results and discussion

**Figure 1.**
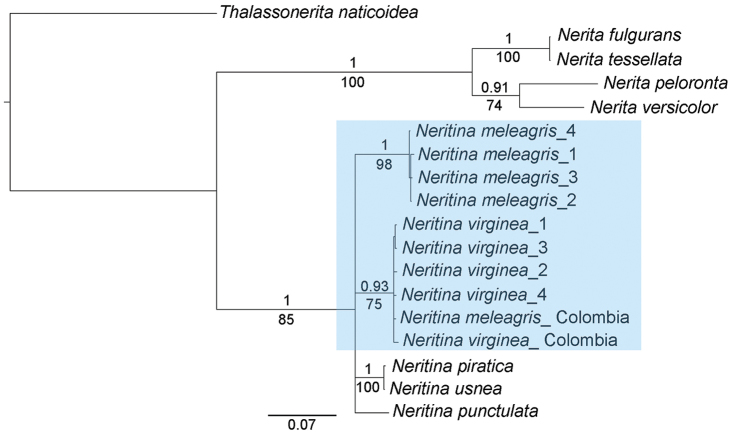
Molecular phylogenetic hypothesis (Bayesian tree) of some species of Neritidae of the Western Atlantic. The Bayesian tree was based on partial mitochondrial COI and 16S sequences. The *Neritina meleagris* and *Neritina virginea* clades (ingroup) are highlighted. The other taxa were used as outgroup. Numbers on and below the main branches represent the posterior Bayesian probabilities (BP) (>0.90) and bootstrap values for maximum likelihood (ML) (>70%), respectively. Specimens with the number “1” are from Camocim beach (Ceará State, NE Brazil) and those with numbers “2”, “3”, and “4” are from Barra Grande beach (Piauí State, NE Brazil). The numbered specimens of *N. virginea* (1, 2, 3, and 4) and *N. meleagris* (1, 2, 3, and 4) are the same specimens shown in Figure 3.

The results of our molecular analyses (phylogeny, species delimitation, and statistical parsimony network) confirmed the existence of two strongly supported clades living in sympatry in the intertidal beachrocks of Northeastern Brazil (Brazilian Province). The Bayesian and maximum likelihood trees showed the same topology, with the formation of four clades within *Neritina*: Group I (*Neritina piratica* + *Neritina usnea*), Group II (*Neritina meleagris*, collected in NE Brazil), Group III (*Neritina virginea*, collected in NE Brazil and Colombia, + “*Neritina meleagris*”, from Colombia), and Group IV (*Neritina punctulata*) (Fig. 1). The Groups II and III were also observed in the ABGD analysis. Intraspecific distances in these groups were at least one order of magnitude smaller than the interspecific distances. Our minimum interspecific genetic distance values (COI region only), involving groups II and III, were 8.4 and 9.6%, respectively (Table 2). These values are higher than the minimum value assumed by Abdou et al. (2017) to characterize distinct Indo-Pacific *Neritina* species. In addition, the probabilities of a new sequence fitting inside P ID (Strict) or at least the sister group P ID (Liberal) of these clades were equal to, or in most cases, greater than 84% (Table 2). These results are compatible with the values found to delimit species in different groups of gastropods (e.g. Churchill et al. 2014; Cooke et al. 2014; Espinoza et al. 2014).

**Table 2.** Species delimitation results from the Bayesian concatenated and Neighbor-Joining trees. These analyses were performed with the Species Delimitation plugin for Geneious.

**Bayesian concatenated tree (COI + 16S)**						
**Species**	**Monophyly**	**Intra Dist**	**Inter Dist-closest**	**Intra/Inter**	**P ID(Strict)**	**P ID(Liberal)**
*Neritina virginea*	yes	0.006	0.073	0.08	0.88 (0.76, 1.0)	0.97 (0.87, 1.0)
*Neritina meleagris*	yes	0.004	0.088	0.04	0.84 (0.70, 0.98)	0.97 (0.86, 1.0)
**Neighbor-Joining K2P COI tree**						
**Species**	**Monophyly**	**Intra dist**	**Inter Dist-closest**	**Intra/Inter**	**P ID(Strict)**	**P ID(Liberal)**
*Neritina virginea*	yes	0.008	0.084	0.09	0.87 (0.75, 1.00)	0.97 (0.87, 1.0)
*Neritina meleagris*	yes	0.002	0.096	0.02	0.86 (0.72, 1.00)	0.98 (0.87, 1.0)

Although the distinction between clades showed high support values, the phylogenetic relationship between them could not be recovered. As we did not have access to the specimens, it was not possible to check the shell colour patterns of the *Neritina meleagris* from Colombia (obtained from GenBank) that was included in the same clade as *Neritina virginea*. Thus, we suspect that an error may have occurred at the time of submission of the sequences to GenBank, since, in the study of Quintero-Galvis and Castro (2013), *N. virginea* and *N. meleagris* appeared in very distinct branches of the phylogenetic tree.

**Figure 2.**
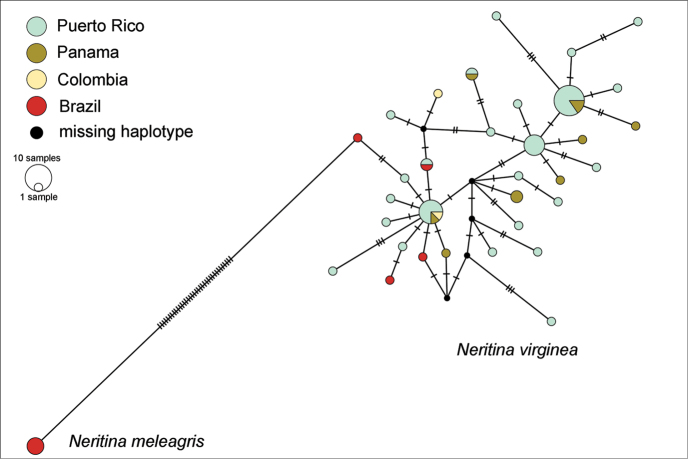
Statistical parsimony network analysis (TCS algorithm) based on 64 partial mitochondrial COI sequences (347 bp). This analysis included specimens of *Neritina meleagris* and *Neritina virginea* from the Caribbean and Brazilian Provinces. Size of the circle is proportional to frequency of the haplotype and colours inside the circles designate geographical locations to which the samples belong. Black circles correspond to hypothetical haplotypes. The number of mutational steps is indicated by dashes on branches. We highlighted the 36 mutational steps that separate the two species haplotypes.

Despite the geographical distance, all *N. virginea* sequences from Brazil and the Caribbean were very similar, with all haplotypes grouped within a few mutational steps (Fig. 2). This result reinforces the validity of *N. virginea* and confirms its presence in the Brazilian Province. As also observed in the phylogenetic analysis, the only sequence assigned as *N. meleagris* from the Caribbean Province is positioned within one of the most frequent *N. virginea* haplotypes for this region (Fig. 2). With respect to our *N. meleagris* sequences, although this species is found in sympatry with its congener in the Brazilian Province, it is separated from the *N. virginea* haplogroups by at least 36 mutational steps.

**Figure 3.**
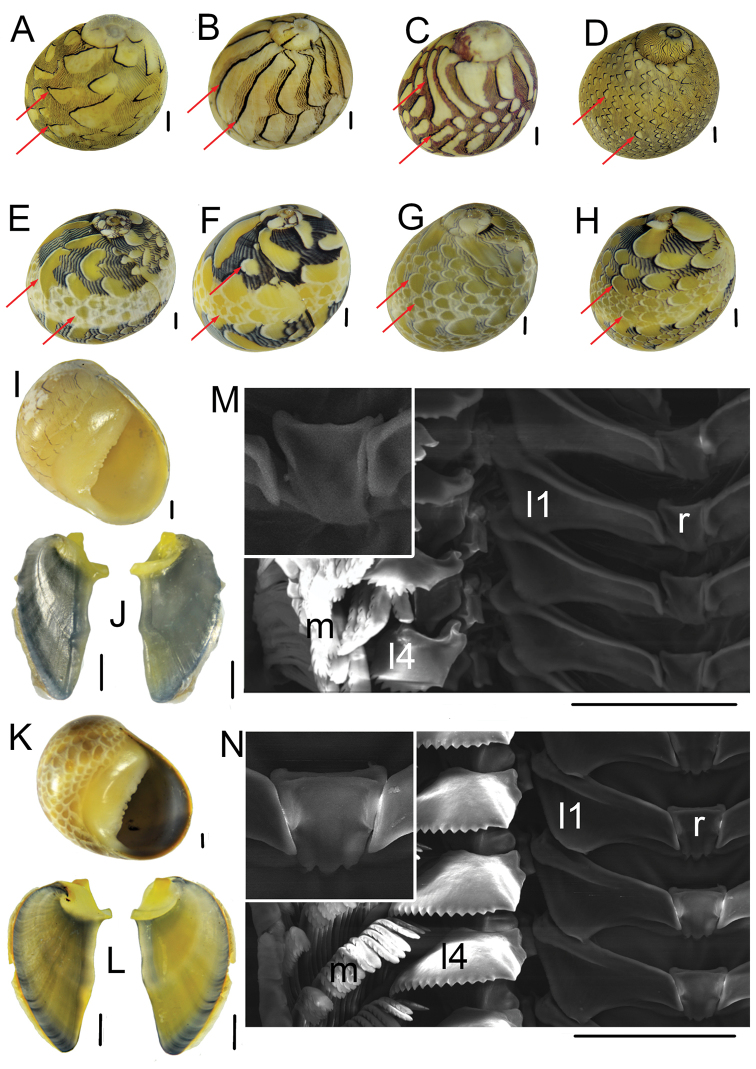
Colour patterns of shells, opercula, and radulae of the *Neritina virginea* and *Neritina meleagris* analysed. The red arrows highlight the differences between the leading edges of colour patterns of both species: *N. virginea* has the leading edges outlined in heavy black, while *N. meleagris* has the leading edge outlined in white or black and white. **A***Neritina virginea*_1 **B***Neritina virginea*_2 **C***Neritina virginea*_3 **D***Neritina virginea*_4 **E***Neritina meleagris*_1 **F***Neritina meleagris*_2 **G***Neritina meleagris*_3 **H***Neritina meleagris*_4 **I** ventral view of shell of *Neritina virginea***J** operculum (outer and inner views) of *Neritina virginea***K** ventral view of shell of *Neritina meleagris***L** operculum (outer and inner views) of *Neritina meleagris***M** radula of *Neritina virginea* (SEM), with rachidian tooth enlarged in the upper left quadrant **N** radula of *Neritina meleagris* (SEM), with rachidian tooth enlarged in the upper left quadrant. Abbreviations: l1 first lateral tooth, l4 fourth lateral tooth, m marginal teeth, r rachidian tooth. The specimens with the number “1” are from Camocim beach (Ceará State, NE Brazil) and those with numbers “2”, “3”, and “4” are from Barra Grande beach (Piauí State, NE Brazil). The numbered specimens of *N. virginea* (1, 2, 3, and 4) and *N. meleagris* (1, 2, 3, and 4) are the same specimens used in the phylogenetic analysis of Figure 1. Scale bars: 1.0 mm (**A–L**); 100 μm (**M, N**).

Figure 3 shows the colour patterns, opercula, and radulae of the *N. meleagris* and *N. virginea* specimens collected from the Barra Grande and Camocim beaches. Our molecular results are consistent with the morphological descriptions previously proposed for each species (Russell 1941; Matthews-Cascon et al. 1990; Eichhorst 2016). Russell (1941) described, for both species, a colour pattern consisting of dark zigzag lines and lighter spots. However, this author emphasized that while *N. virginea* has a leading edge outlined in heavy black, *N. meleagris* instead has a leading edge outlined with white, white and black, or white and red, resembling imbricating scales. The imbricating scales pattern was emphasized in the original description of *N. meleagris* (Lamarck 1822), whereas Matthews-Cascon et al. (1990) and Eichhorst (2016) highlighted the differences in the leading edges of the colour pattern for each species. Although did not examine the type specimens, individuals of *N. virginea* from the Linnean Collection at the Natural History Museum, London (see http://linnean-online.org/17152/), and *N. meleagris*, from the type locality (Dominican Republic) (see http://data.biodiversitydata.nl/naturalis/specimen/ZMA.MOLL.313038), had the same leading edge patterns as described earlier. All analysed specimens of *N. meleagris* had the leading edge outlined with white or white and black, while *N. virginea* specimens had the leading edge outlined in black (Fig. 3A–H). Despite the great variation in their shell colour patterns, a more detailed observation of the leading edges of the *N. virginea* and *N. meleagris* shells allows the separation of the two species, even in the field. Warmke and Abbott (1962) also emphasized the use of leading edges to separate the two species. Williams (2017) argued that the colours and patterns of gastropod shells could be genetically determined, influenced directly by environmental factors, or a combination of both. Specifically, the patterns of leading edges (leading edge outlined with white or white and black in *N. meleagris* and outlined in black in *N. virginea*) appear to be under genetic control rather than be influenced directly by environmental factors, since the patterns for each species are consistent regardless of the location studied (e.g. Russell 1941; Eichhorst 2016; present study). This observation is reinforced by the clades obtained in our phylogenetic analysis, corroborating the diagnostic colour patterns previously described (Fig. 1).

Besides the shell colour patterns, *N. virginea* and *N. meleagris* differ from each other in subtle ways. The inner lips of the shells of the two species are denticulated. However, in *N. virginea* there are several small denticles interspersed by two larger teeth, while in *N. meleagris* the teeth are larger, more prominent in the central region, and less numerous when compared to *N. virginea* (Fig. 3I, K). Russell (1941), Matthews-Cascon et al. (1990), and Eichhorst (2016) also highlighted these differences regarding the number of teeth on the inner lip. Both species have a calcareous and smooth operculum, with a bifurcated apophysis. Comparing the opercula, *N. virginea* has a darker (bluish-black) and more elongated operculum, with the apophysis elements thinner and more separated from each other. On the other hand, the operculum of *N. meleagris* presents a lighter coloration (yellowish-black) and a semi-circular shape, with the apophysis elements much stouter and closer to each other (Fig. 3J, L). In the present study, *Neritina virginea* and *N. meleagris* have a very similar morphology of the radula: a rhipidoglossate radula, with one rachidian tooth, five pairs of lateral teeth, and many denticulated marginal teeth arranged in transverse rows (Fig. 3M, N; see also Suppl. material 2). The most striking difference between these radulae is the rectangular rachidian tooth, which has three cusps in *N. meleagris* (both male and female) but is cuspless in *N. virginea*. The first lateral tooth of *N. virginea* is more slender than that of *N. meleagris*. Previous studies have shown that the radula teeth pattern of neritids is very stable, the most variable character being the number of cusps on the fifth lateral tooth, which is likely correlated with age (Baker 1923; Huang 1997; Haynes 2001). This characteristic makes it difficult to define intra- and interspecific differences. Further studies are needed to better define the differences between the radulae of the two species.

Our molecular data show that *N. virginea* and *N. meleagris* are two distinct species, thus confirming the *N. meleagris* record for the Brazilian coast. In summary, our results, along with the already well-established record of *Neritina zebra* (Matthews-Cascon et al. 1990; Rios 2009; Barroso et al. 2012, 2016; Eichhorst 2016), demonstrate that there are three species of the genus *Neritina* registered for the Brazilian Province to date. We emphasize the importance of the separation of *N. virginea* and *N. meleagris* in future studies, especially those conducted in the Brazilian Province, since these species may cohabit. In the field, these species can be identified with a detailed observation of the leading edge patterns of their shells, assisting ecological studies. Further research is needed in other areas along the Brazilian Province to determine the geographic distribution of *N. virginea* and *N. meleagris*, highlighting the locations where they co-occur.

## Supplementary materials

Supplementary material 1 Alignments used to construct the phylogenetic trees and statistical parsimony network analysis https://binary.pensoft.net/file/372782This dataset is made available under the Open Database License (http://opendatacommons.org/licenses/odbl/1.0/). The Open Database License (ODbL) is a license agreement intended to allow users to freely share, modify, and use this Dataset while maintaining this same freedom for others, provided that the original source and author(s) are credited.Cristiane Xerez Barroso, João Eduardo Pereira de Freitas, Helena Matthews-Cascon, Luis Ernesto Arruda Bezerra, Tito Monteiro da Cruz Lotufo

Supplementary material 2 Radulae of the *Neritina meleagris* (A) and *Neritina virginea* (B) analysed https://binary.pensoft.net/file/372783This dataset is made available under the Open Database License (http://opendatacommons.org/licenses/odbl/1.0/). The Open Database License (ODbL) is a license agreement intended to allow users to freely share, modify, and use this Dataset while maintaining this same freedom for others, provided that the original source and author(s) are credited.Cristiane Xerez Barroso, João Eduardo Pereira de Freitas, Helena Matthews-Cascon, Luis Ernesto Arruda Bezerra, Tito Monteiro da Cruz Lotufo
