# Supplementary material for: Molecular evidences confirm the taxonomic separation of two sympatric congeneric species (Mollusca, Gastropoda, Neritidae, Neritina)
Source: Zookeys. 2020 Jan 16;904:117–30. doi: 10.3897/zookeys.904.46790 (PMC6978406; doi:10.3897/zookeys.904.46790)
Supplement: Supplementary material 1 [file zookeys-904-117-s001.pdf]

Molecular evidences confirm the taxonomic separation of two sympatric congeneric species (Mollusca, Gastropoda, Neritidae, *Neritina*)

Cristiane Xerez Barroso[1,2], João Eduardo Pereira de Freitas[3], Helena Matthews-Cascon[1,2], Luis Ernesto Arruda Bezerra[1,4] & Tito Monteiro da Cruz Lotufo[5]

Supplementary file _1 - Alignments used to construct the phylogenetic trees and statistical parsimony network analysis.

Alignment used to construct the phylogenetic trees
>Neritina_meleagris_4
ACTTTGTATATTATGTTTGGTGTTTGATCTGGTCTAGTAGGAACTGCTCTTAGTTTACTAAT
TCGTGCTGAGCTTGGACAGCCTGGTGCTTTATTAGGGGATGACCAGTTATATAATGTTATT
GTAACTGCTCATGCATTTGTAATAATTTTCTTTTTGGTGATGCCAATGATGATTGGTGGTTT
TGGGAACTGATTAGTGCCTTTAATGTTAGGGGCTCCTGATATGGCTTTTCCTCGGTTGAAT
AACATAAGTTTTTGACTTCTCCCTCCATCATTAACTTTATTACTAGCTTCTTCTGCTGTTGA
AAGTGGGGTGGGAACTGGTTGAACTGTTTATCCTCCACTTTCAGGTAATCTTGCTCATGCT
GGTGGGTCTGTTGATTTAGCTATTTTTTCTTTGCATTTAGCGGGTGTTTCCTCAATCCTAGG
TGCTGTAAACTTTATTACTACAATTATTAACATGCGATGACAGGGGATGCAGTTTGAACGA
TTGCCATTGTTTGTTTGATCTGTAAAGATTACTGCAATTTTGTTATTACTTTCTCTTCCTGTG
TTAGCAGGGGCAATTACTATATTATTGACTGATCGAAACTTTAATACTTCATTTTTTGATCC
AGCGGGAGGGGGAGATCCAATCCAGTGATTGCTTTTTAACGGCCGCGGTACCCTGACCGT
GCAAAGGTAGCATAATCATTTGCCTTTTAATTGAAGGCTGGTATGAACGGTTTGACGAGA
GTAAAGCTGTCTCACATATATTCAATAAAAATTAATTTAAAGGTGCAAAAGCCTTTATTAC
CCAAAAAGACGAGAAGACCCTGTTGAGCTTTAATTTAACTTAAGTACTAACTATATATTAA
TATAATTCACTTACTTGATAAAAATTTTAATTGGGGCGATTAAGGAACACTCAAAAGCTTC
CTGACCACTA-
AAATAAAATATTCCATAGATTAAATCGATCCAGCAATGCTGATCAACAAAATGAGTTACC
ACAGGGATAACAGCATAATTCTTTTTGAGAGCTCATATCGAAAAAAGAGATTGTGACCTC
GATGTTGGACTAGGGTAACCGG-AAGGTGCAGCCGC-TTTCCCGCT-TG-
GTCTGTTCGACCAATAATTCCCT
>Neritina_meleagris_1
ACTTTGTATATTATGTTTGGTGTTTGATCTGGTCTAGTAGGAACTGCTCTTAGTTTACTAAT
TCGTGCTGAGCTTGGACAGCCTGGTGCTTTATTAGGGGATGACCAGTTATATAATGTTATT
GTAACTGCTCATGCATTTGTAATAATTTTCTTTTTGGTGATGCCAATGATGATTGGTGGTTT
TGGGAATTGATTAGTGCCTTTAATGTTAGGGGCTCCTGATATGGCTTTTCCTCGGTTGAAT
AACATAAGTTTTTGACTTCTCCCTCCATCATTAACTTTATTACTAGCTTCTTCTGCTGTTGA
AAGTGGGGTGGGAACTGGTTGAACTGTTTATCCTCCACTTTCAGGTAATCTTGCTCATGCT
GGTGGGTCTGTTGATTTAGCTATTTTTTCTTTGCATTTAGCGGGTGTTTCCTCAATCCTAGG
TGCTGTAAACTTTATTACTACAATTATTAACATGCGATGACAGGGGATGCAGTTTGAACGA
TTGCCATTGTTTGTTTGATCTGTAAAGATTACTGCAATTTTGTTATTACTTTCTCTTCCTGTG
TTAGCAGGGGCAATTACTATATTATTGACTGATCGAAACTTTAATACTTCATTTTTTGATCC
AGCGGGAGGGGGAGATCCAATCCAGTGATTGCTTTTTAACGGCCGCGGTACCCTGACCGT
GCAAAGGTAGCATAATCATTTGCCTTTTAATTGAAGGCTGGTATGAACGGTTTGACGAGA
GTAAAGCTGTCTCACATATATTCAATAAAAATTAATTTAAAGGTGCAAAAGCCTTTATTAC
CCAAAAAGACGAGAAGACCCTGTTGAGCTTTAATTTAACTTAAGTACTAACTATATATTAA
TATAATTCACTTACTTAATAAAAATTTTAATTGGGGCGATTAAGGAACACGCAAAAGCTTC
CTGACTACTA-
AAATAAAATATTCCATAGATTAAATCGATCCAGCAATGCTGATCAACAAAATGAGTTACC
ACAGGGATAACAGCATAATTCTTTTTGAGAGCTCATATCGAAAAAAGAGATTGTGACCTC
GATGTTGGACTAGGGTAACCGG-AAGGTGCAGCCGC-TTTCCCGCT-TG-
GTCTGTTCGACCAATAATTCCCT
>Neritina_meleagris_3
ACTTTGTATATTATGTTTGGTGTTTGATCTGGTCTAGTAGGAACTGCTCTTAGTTTACTAAT
TCGTGCTGAGCTTGGACAGCCTGGTGCTTTATTAGGGGATGACCAGTTATATAATGTTATT
GTAACTGCTCATGCATTTGTAATAATTTTCTTTTTGGTGATGCCAATGATGATTGGTGGTTT
TGGGAACTGATTAGTGCCTTTAATGTTAGGGGCTCCTGATATGGCTTTTCCTCGGTTGAAT
AACATAAGTTTTTGACTTCTCCCTCCATCATTAACTTTATTACTAGCTTCTTCTGCTGTTGA
AAGTGGGGTGGGAACTGGTTGAACTGTTTATCCTCCACTTTCAGGTAATCTTGCTCATGCT
GGTGGGTCTGTTGATTTAGCTATTTTTTCTTTGCATTTAGCGGGTGTTTCCTCAATCCTAGG
TGCTGTAAACTTTATTACTACAATTATTAACATGCGATGACAGGGGATGCAGTTTGAACGA

TTGCCATTGTTTGTTTGATCTGTAAAGATTACTGCAATTTTGTTATTACTTTCTCTTCCTGTG
TTAGCAGGGGCAATTACTATATTATTGACTGATCGAAACTTTAATACTTCATTTTTGACCC
AGCGGGAGGGGGAGATCCAATCCAGTGATTGCTTTTTAACGGCCGCGGTACCCTGACCGT
GCAAAGGTAGCATAATCATTTGCCTTTTAATTGAAGGCTGGTATGAACGGTTTGACGAGA
GTAAAGCTGTCTCACATATATTCAATAAAAATTAATTTAAAGGTGCAAAAGCCTTTATTAC
CCAAAAAGACGAGAAGACCCTGTTGAGCTTTAATTTAACTTAAGTACTAACTATATATTAA
TATAATTCACTTACTTAATAAAAATTTTAATTGGGGCGATTAAGGAACACTCAAAAGCTTC
CTGACCACTA-
AAATAAAATATTCCATAGATTAAATCGATCCAGCAATGCTGATCAACAAAATGAGTTACC
ACAGGGATAACAGCATAATTCTTTTTGAGAGCTCATATCGAAAAAAGAGATTGTGACCTC
GATGTTGGACTAGGGTAACCGG-AAGGTGCAGCCGC-TTTCCCGCT-TG-
GTCTGTTCGACCAATAATTCCCT
>Neritina_meleagris_2
ACTTTGTATATTATGTTTGGTGTTTGATCTGGTCTAGTAGGAACTGCTCTTAGTTTACTAAT
TCGTGCTGAGCTTGGACAGCCTGGTGCTTTATTAGGGGATGACCAGTTATATAATGTTATT
GTAACTGCTCATGCATTTGTAATAATTTTCTTTTTGGTGATGCCAATGATGATTGGTGGTTT
TGGGAACTGATTAGTGCCTTTAATGTTAGGGGCTCCTGATATGGCTTTTCCTCGGTTGAAT
AACATAAGTTTTTGACTTCTCCCTCCATCATTAACTTTATTACTAGCTTCTTCTGCTGTTGA
AAGTGGGGTGGGAACTGGTTGAACTGTTTATCCTCCACTTTCAGGTAATCTTGCTCATGCT
GGTGGGTCTGTTGATTTAGCTATTTTTTCTTTGCATTTAGCGGGTGTTTCCTCAATCCTAGG
TGCTGTAAACTTTATTACTACAATTATTAACATGCGATGACAGGGGATGCAGTTTGAACGA
TTGCCATTGTTTGTTTGATCTGTAAAGATTACTGCAATTTTGTTATTACTTTCTCTTCCTGTG
TTAGCAGGGGCAATTACTATATTATTGACTGATCGAAACTTTAATACTTCATTTTTGATCC
AGCGGGAGGGGGAGATCCAATCCAGTGATTGCTTTTTAACGGCCGCGGTACCCTGACCGT
GCAAAGGTAGCATAATCATTTGCCTTTTAATTGAAGGCTGGTATGAACGGTTTGACGAGA
GTAAAGCTGTCTCACATATATTCAATAAAAATTAATTTAAAGGTGCAAAAGCCTTTATTAC
CCAAAAAGACGAGAAGACCCTGTTGAGCTTTAATTTAACTTAAGTACTAACTATATATTAA
TATAATTCACTTACTTAATAAAAATTTTAATTGGGGCGATTAAGGAACACTCAAAAGCTTC
CTGACCACTA-
AAATAAAATATTCCATAGATTAAATCGATCCAGCAATGCTGATCAACAAAATGAGTTACC
ACAGGGATAACAGCATAATTCTTTTTGAGAGCTCATATCGAAAAAAGAGATTGTGACCTC
GATGTTGGACTAGGGTAACCGG-AAGGTGCAGCCGC-TTTCCCGCT-TG-
GTCTGTTCGACCAATAATTCCCT
>Neritina_virginea_1
ACTTTGTATATTATGTTTGGTGTTTGATCTGGTCTGGTAGGAACCGCTCTTAGTTTGTTAAT
TCGAGCTGAGCTTGGACAGCCTGGTGCTTTACTAGGGGATGACCAGCTATACAATGTAATT
GTTACTGCTCATGCATTTGTAATAATTTTCTTTTTGGTGATGCCAATGATGATTGGTGGATT
TGGAAACTGATTGGTGCCTCTAATGTTAGGAGCTCCTGATATGGCTTTTCCTCGGCTAAAT
AACATAAGTTTTTGGCTTCTTCCTCCATCATTAACTTTGCTATTAGCTTCTTCTGCCGTTGA
AAGTGGGGTAGGGACTGGTTGAACTGTTTATCCTCCGCTTTCAGGTAATTTAGCTCATGCG
GGTGGGTCTGTTGACTTAGCAATTTTTTCTTTACATTTAGCAGGTGTTTCTTCAATTTTAGG
TGCTGTGAATTTTATTACCACAATTATTAATATGCGATGACAGGGTATGCAATTTGAACGA
TTACCATTATTTGTTTGATCTGTAAAAATTACTGCAATTCTGTTGTTGCTTTCTCTTCCTGTG
CTAGCAGGTGCAATTACTATGCTGTTAACTGATCGAAACTTCAATACTTCATTCTTTGATC
CAGCAGGAGGGGGTGATCCAATCCAGTGATTGCTTTTTAACGGCCGCGGTACCCTGACCG
TGCAAAGGTAGCATAATCATTTGCCTTTTAATTGAAGGCTGGTATGAACGGTTTGACGAGA
GTAAAGCTGTCTCACATATACTTAATAAAAATTAATTTAAAGGTGCAAAAGCCTTTATTAC
CCAAAAAGACGAGAAGACCCTGTTGAGCTTTAATTTAGCCCAAGTATTAACTATATATTA
ATATAATTCACTTACTCGGTAAAAATTTTAATTGGGGCGATTAAGGAACACACAAAAGCT
TCCTGATTATT--
CAGTAAAATATTCAATAGATTAAATCGATCCAGCAATGCTGATCAACAAAATGAGTTACC
ACAGGGATAACAGCATAATTCTTTTTGAGAGCTCATATCGAAAAAAGAGATTGTGACCTC
GATGTTGGACTAGGGTAACCGG-AAGGTGCAGCCGC-TTTCCTGCT-TG-
GTCTGTTCGACCAATAATTCCCT
>Neritina_virginea_2
ACTTTGTATATTATGTTTGGTGTTTGATCTGGTCTGGTAGGAACCGCTCTTAGTTTGTTAAT
TCGAGCTGAGCTTGGACAGCCTGGTGCTTTACTAGGGGATGACCAGCTATATAATGTAATT
GTTACTGCTCATGCATTTGTAATAATTTTCTTTTTGGTGATGCCAATGATGATTGGTGGATT
TGGAAACTGATTGGTGCCTCTAATGTTAGGAGCTCCTGATATGGCTTTTCCTCGGCTAAAT
AACATAAGTTTTTGGCTTCTCCCTCCATCATTAACTTTGCTATTAGCTTCTTCTGCCGTTGA

AAGTGGGGTAGGAACTGGTTGAACTGTTTATCCTCCGCTTTCAGGTAATTTAGCTCATGCG
GGTGGGTCTGTTGACTTAGCAATTTTTCTTTACATTTAGCAGGTGTTTCTTCAATTTTAGG
TGCTGTGAATTTTATTACCACAATTATTAATATGCGATGACAGGGTATGCAATTTGAACGA
TTACCATTATTTGTTTGATCTGTAAAAATTACTGCAATTCTGTTGTTGCTTTCTCTTCCTGTG
CTAGCAGGTGCAATTACTATGCTGTTAACTGATCGAAACTTCAACACTTCATTTTTTGATC
CAGCAGGAGGGGGTGATCCAATCCAGTGATTGCTTTTTAACGGCCGCGGTACCCTGACCG
TGCAAAGGTAGCATAATCATTTGCCTTTTAATTGAAGGCTGGTATGAACGGTTTGACGAGA
GTAAAGCTGTCTCACATATACTTAATAAAAATTAATTTAAAGGTGCAAAAGCCTTTATTAC
CCAAAAAGACGAGAAGACCCTGTTGAGCTTTAATTTAGCCCAAGTATTAACTATATATTA
ATATAATTCACTTACTCGGTAAAAATTTTAATTGGGGCGATTAAGGAACACACAAAAGCT
TCCTGATTATT--
CAGTAAAATATTCAATAGATTAAATCGATCCAGCAATGCTGATCAACAAAATGAGTTACC
ACAGGGATAACAGCATAATTCTTTTTGAGAGCTCATATCGAAAAAAGAGATTGTGACCTC
GATGTTGGACTAGGGTAACCGG-AAGGTGCAGCCGC-TTTCCTGCT-TG-
GTCTGTTCGACCAATAATTCCCT
>Neritina_virginea_4
ACTTTGTATATTATGTTTGGTGTTTGATCTGGTCTGGTAGGAACCGCTCTTAGTTTGTTAAT
TCGAGCTGAGCTTGGACAGCCTGGTGCTTTACTAGGGGATGACCAGCTATATAATGTAATT
GTTACTGCTCATGCATTTGTAATAATTTTCTTTTTGGTGATGCCAATGATGATTGGTGGATT
TGGAAACTGATTGGTGCCTCTAATGTTAGGAGCTCCTGATATGGCTTTTCCTCGGCTAAAT
AACATAAGTTTTTGGCTTCTTCCACCATCATTAACTTTGCTATTAGCTTCTTCTGCCGTTGA
AAGTGGGGTAGGGACTGGTTGAACTGTTTATCCTCCGCTTTCAGGTAATTTAGCTCATGCG
GGTGGGTCTGTTGACTTAGCAATTTTTCTTTACATTTAGCAGGTGTTTCTTCAATTTTAGG
TGCTGTGAATTTTATTACCACAATTATTAATATGCGATGACAGGGTATGCAATTTGAACGA
TTACCATTATTTGTTTGATCTGTAAAAATTACTGCAATTCTGTTGCTGCTTTCTCTTCCTGTG
CTAGCAGGTGCAATTACTATGCTGTTAACTGATCGAAACTTCAACACTTCATTCTTTGATC
CAGCAGGAGGGGGTGATCCAATCCAGTGATTGCTTTTTAACGGCCGCGGTACCCTGACCG
TGCAAAGGTAGCATAATCATTTGCCTTTTAATTGAAGGCTGGTATGAACGGTTTGACGAGA
GTAAAGCTGTCTCACATATACTTAATAAAAATTAATTTAAAGGTGCAAAAGCCTTTATTAC
CCAAAAAGACGAGAAGACCCTGTTGAGCTTTAATTTAGCCCAAGTATTAACTATATATTA
ATATAATTCACTTACTCGGTAAAAATTTTAATTGGGGCGATTAAGGAACACACAAAAGCT
TCCTGATTATT--
CAGTAAAATATTCAATAGATTAAATCGATCCAGCAATGCTGATCAACAAAATGAGTTACC
ACAGGGATAACAGCATAATTCTTTTTGAGAGCTCATATCGAAAAAAGAGATTGTGACCTC
GATGTTGGACTAGGGTAACCGG-AAGGTGCAGCCGC-TTTCCTGCT-TG-
GTCTGTTCGACCAATAATTCCCT
>Neritina_meleagris_Colombia
ACTTTGTATATTATGTTTGGTGTTTGATCTGGTCTGGTAGGAACCGCTCTTAGTTTGTTAAT
TCGAGCTGAGCTTGGACAGCCTGGTGCTCTACTAGGGGATGACCAGCTATATAATGTAATT
GTTACTGCTCATGCATTTGTAATAATTTTCTTTTTGGTGATGCCAATGATGATTGGTGGATT
TGGAAACTGATTGGTGCCTCTAATGTTAGGAGCTCCTGATATGGCTTTTCCTCGGCTAAAT
AACATAAGTTTTTGGCTTCTTCCTCCATCATTAACTTTGCTATTAGCTTCTTCTGCCGTTGA
AAGTGGGGTAGGGACTGGTTGAACTGTTTATCCTCCGCTTTCAGGTAATTTAGCTCATGCG
GGTGGGTCTGTTGACTTAGCAATTTTTCTTTACATTTAGCAGGTGTTTCTTCAATTTTAGG
TGCTGTGAATTTTATTACCACAATTATTAATATGCGATGACAGGGTATGCAATTTGAACGA
TTACCATTATTTGTTTGATCTGTAAAAATTACTGCAATTCTGTTGTTGCTTTCTCTTCCTGTG
CTAGCAGGTGCAATTACTATGCTGTTAACTGATCGAAACTTCAACACTTCATTCTTTGATC
CAGCAGGAGGGGGTGATCCAATCCAGTGATTGCTTTTTAACGGCCGCGGTACCCTGACCG
TGCAAAGGTAGCATAATCATTTGCCTTTTAATTGAAGGCTGGTATGAACGGTTTGACGAGA
GTAAAGCTGTCTCACATATACTTAATAAAAATTAATTTAAAGGTGCAAAAGCCTTTATTAC
CCAAAAAGACGAGAAGACCCTGTTGAGCTTTAATTTAGCCCAAGTATTAACTATATATTA
ATATAATTCACTTACTCGGTAAAAATTTTAATTGGGGCGATTAAGGAACACACAAAAGCT
TCCTGATTATT--
CAATAAAATATTCAATAGATTAAATCGATCCAGCAATGCTGATCAACAAAATGAGTTACC
ACAGGGATAACAGCATAATTCTTTTTGAGAGCTCATATCGAAAAAAGAGATTGTGACCTC
GATGTTGGACTAGGGTAACCGG-AAGGTGCAGCCGCTTTTCCTGCT-TG-
GTCTGTTCGACCAATAATTCCCT
>Neritina_virginea_Colombia
ACTTTGTATATTATGTTTGGTGTTTGATCTGGTCTGGTAGGAACCGCTCTTAGTTTGTTAAT
TCGAGCTGAGCTTGGACAGCCTGGTGCTTTACTAGGGGATGACCAGTTATATAATGTAATT

GTTACTGCTCATGCATTTGTAATAATTTTCTTTTTGGTGATGCCAATGATGATTGGTGGATT
TGGAAACTGATTGGTGCCTCTAATGTTAGGAGCTCCTGATATGGCTTTTCCTCGGCTAAAT
AACATAAGTTTTTGGCTCCTTCCTCCATCACTAACTTTGCTATTAGCTTCTTCTGCCGTTGA
AAGTGGGGTAGGGACTGGTTGAACTGTTTATCCTCCGCTTTCAGGTAATTTAGCTCATGCG
GGTGGGTCTGTTGACTTAGCAATTTTTTCTTTGCATTTAGCAGGTGTTTCTTCAATTTTAGG
TGCTGTGAATTTTATCACCACAATTATTAATATGCGATGACAGGGTATGCAATTTGAACGA
TTACCATTATTTGTTTGATCTGTAAAAATTACTGCAATTCTGTTGTTGCTTTCTCTTCCTGTG
CTAGCAGGTGCAATTACTATGCTGTTAACTGATCGAAACTTCAACACTTCATTCTTTGATC
CAGCAGGAGGGGGTGATCCAATCCAGTGATTGCTTTTTAACGGCCGCGGTACCCTGACCG
TGCAAAGGTAGCATAATCATTTGCCTTTTAATTGAAGGCTGGTATGAACGGTTTGACGAGA
GTAAAGCTGTCTCACATATACTTAATAAAAATTAATTTAAAGGTGCAAAAGCCTTTATTAC
CCAAAAAGACGAGAAGACCCTGTTGAGCTTTAATTTAGCCCAAGTATTAACTATATATTA
ATATAATTCACTTACTCGGTAAAAATTTTAATTGGGGCGATTAAGGAACACACAAAAGCT
TCCTGATTATT--
CAGTAAAATATTCAATAGATTAAATCGATCCAGCAATGCTGATCAACAAAATGAGTTACC
ACAGGGATAACAGCATAATTCTTTTTGAGAGCTCATATCGAAAAAAGAGATTGTGACCTC
GATGTTGGACTAGGGTAACCGG-AAGGTGCAGCCGC-TTTCCTGCT-TG-
GTCTGTTCGACCAATAATTCCCT
>Neritina_virginea_3
ACTTTGTATATTATGTTTGGTGTTTGATCTGGTCTGGTAGGAACCGCTCTTAGTTTGTTAAT
TCGAGCTGAGCTTGGACAGCCTGGTGCTTTACTAGGGGATGACCAGCTATACAATGTAATT
GTTACTGCTCATGCATTTGTAATAATTTTCTTTTTGGTGATGCCAATGATGATTGGTGGATT
TGGAAACTGATTGGTGCCTCTAATGTTAGGAGCTCCTGATATGGCTTTTCCTCGGCTAAAT
AACATAAGTTTTTGGCTTCTTCCTCCATCATTAACTCTGCTATTAGCTTCTTCTGCCGTTGA
AAGTGGGGTAGGGACTGGTTGAACTGTTTATCCTCCGCTTTCAGGTAATTTAGCTCATGCG
GGTGGGTCTGTTGACTTAGCAATTTTTTCTTTACATTTAGCAGGTGTTTCTTCAATTTTAGG
TGCTGTGAATTTTATTACCACAATTATTAATATGCGATGACAGGGTATGCAATTTGAACGA
TTACCATTATTTGTTTGATCTGTAAAAATTACTGCAATTCTGTTGTTGCTTTCTCTTCCTGTG
CTAGCAGGTGCAATTACTATGCTGTTAACTGATCGAAACTTCAACACTTCATTCTTTGATC
CAGCAGGAGGGGGTGATCCAATCCAGTGATTGCTTTTTAACGGCCGCGGTACCCTGACCG
TGCAAAGGTAGCATAATCATTTGCCTTTTAATTGAAGGCTGGTATGAACGGTTTGACGAGA
GTAAAGCTGTCTCACATATACTTAATAAAAATTAATTTAAAGGTGCAAAAGCCTTTATTAC
CCAAAAAGACGAGAAGACCCTGTTGAGCTTTAATTTAGCCCAAGTATTAACTATATATTA
ATATAATTCACTTACTCGGTAAAAATTTTAATTGGGGCGATTAAGGAACACACAAAAGCT
TCCTGATTATT--
CAGTAAAATATTCAATAGATTAAATCGATCCAGCAATGCTGATCAACAAAATGAGTTACC
ACAGGGATAACAGCATAATTCTTTTTGAGAGCTCATATCGAAAAAAGAGATTGTGACCTC
GATGTTGGACTAGGGTAACCGG-AAGGTGCAGCCGC-TTTCCTGCT-TG-
GTCTGTTCGACCAATAATTCCCT
>Neritina_piratica
ACTTTGTATATTATGTTTGGTGTTTGATCTGGGTTGGTAGGAACTGCTCTTAGTTTGTTAAT
TCGAGCTGAGCTTGGACAGCCTGGTGCTTTATTAGGGGATGATCAGTTATATAATGTAATT
GTGACTGCCCATGCATTTGTAATAATTTTCTTTTTGGTAATGCCAATGATAATTGGTGGGTT
TGGAAATTGATTGGTGCCCTTAATGCTAGGGGCTCCTGACATGGCTTTCCCTCGGTTAAAT
AACATAAGTTTTTGACTTCTTCCTCCGTCATTGACTCTGTTATTGGCTTCTTCTGCTGTTGA
GAGTGGGGTAGGAACTGGTTGAACTGTTTATCCTCCACTTTCAGGTAATTTAGCTCATGCA
GGTGGGTCTGTCGACTTGGCTATTTTTTCTTTGCATTTAGCAGGTGTTTCTTCAATTTTAGG
TGCTGTAAATTTTATTACTACAATTATTAATATGCGATGACAAGGAATGCAGTTTGAACGA
TTACCATTGTTTGTTTGATCTGTGAAAATTACTGCAATTTTGTTGTTGCTTTCTCTTCCTGTG
TTAGCAGGCGCAATTACTATGTTGTTGACTGATCGAAATTTTAATACTTCGTTCTTTGATCC
TGCAGGTGGGGGTGATCCAATTCAGTGATTGCTTTTTAACGGCCGCGGTACCCTGACCGTG
CAAAGGTAGCATAATCATTTGCCTTTTAATTGAAGGCTGGTATGAACGGTTTGACGAGAGT
AAAGCTGTCTCACATATACTTAATAAAAATTAATTTAAAGGTGCAAAAGCCTTTATTGCCC
AAAAAGACGAGAAGACCCTGTTGAGCTTTAATTTAACTCAAGTACTAACTATATATTAAT
ATAATTCACTTGCTTGATAAAAATTTTAATTGGGGCGATTAAGGAACATTCAAAAGCTTCC
TGACTATTAACAATAAAATATTCCATAGATTAAATCGATCCAGCAATGCTGATCAACAAA
ATGAGTTACCACAGGGATAACAGCATAATTCTTTTTGAGAGCTCATATCGAAAAAAGAGA
TTGTGACCTCGATGTTGGACTAGGGTAACCGG-AAGGTGCAGCCGC-TTTCCCGCT-
TGCGTCTGTTCGCCCAATAATTCCCT
>Neritina_usnea

ACTTTGTATATTATGTTTGGTGTTTGATCTGGGTTGGTAGGAACTGCTCTTAGTTTGTTAAT
TCGAGCTGAGCTTGGACAGCCTGGTGCTTTATTAGGGGATGATCAGTTATATAATGTAATT
GTGACTGCCCATGCATTTGTAATAATTTTCTTTTTGGTAATGCCAATGATAATTGGTGGGTT
TGGAAATTGATTGGTGCCCTTAATGCTAGGGGCTCCTGACATGGCTTTCCCTCGGTTAAAT
AACATAAGTTTTTGACTTCTTCCTCCGTCATTGACTCTGTTATTGGCTTCTTCTGCTGTTGA
GAGTGGGGTAGGAACTGGTTGAACTGTTTATCCTCCACTTTCAGGTAATTTAGCTCATGCA
GGTGGGTCTGTCGACTTGGCTATTTTTTCTTTGCATTTAGCAGGTGTTTCTTCAATTTTAGG
TGCTGTAAATTTTATTACTACAATTATTAATATGCGATGACAAGGAATGCAGTTTGAACGA
TTACCATTGTTTGTTTGATCTGTGAAAATTACTGCAATTTTGTTGTTGCTTTCTCTTCCTGTG
TTAGCAGGCGCAATTACTATGTTGTTGACTGATCGAAATTTTAATACTTCGTTCTTTGATCC
TGCAGGTGGGGGTGATCCAATTCAGTGATTGCTTTTTAACGGCCGCGGTACCCTGACCGTG
CAAAGGTAGCATAATCATTTGCCTTTTAATTGAAGGCTGGTATGAACGGTTTGACGAGAGT
AAAGCTGTCTCACATATACTTAATAAAAATTAATTTAAAGGTGCAAAAGCCTTTATTGCCC
AAAAAGACGAGAAGACCCTGTTGAGCTTTAATTTAACTCAAGTACTAACTATATATTAAT
ATAATTCACTTGCTTGATAAAAATTTTAATTGGGGCGATTAAGGAACATTCAAAAGCTTCC
TGACTATTAACAATAAAATATTCCATAGATTAAATCGATCCAGCAATGCTGATCAACAAA
ATGAGTTACCACAGGGATAACAGCATAATTCTTTTTGAGAGCTCATATCGAAAAAAGAGA
TTGTGACCTCGATGTTGGACTAGGGTAACCGG-AAGGTGCAGCCGC-TTTCCCGCT-TG-
GTCTGTTCGACCAATAATTCCCT
>Neritina_punctulata
ACTTTGTATATTATGTTTGGTGTTTGATCTGGTCTGGTAGGAACTGCTCTTAGTTTGTTGAT
TCGAGCTGAGCTTGGACAGCCTGGTGCTCTATTGGGAGATGACCAGTTATACAATGTAATT
GTAACTGCTCATGCATTTGTAATAATTTTCTTTTTGGTAATGCCAATGATAATTGGTGGATT
TGGAAACTGACTGGTGCCTTTAATGTTAGGGGCTCCTGACATGGCTTTCCCTCGATTGAAT
AATATAAGTTTTTGACTTCTTCCTCCATCATTGACTTTACTACTAGCCTCTTCTGCTGTTGA
GAGTGGGGTAGGGACTGGTTGAACTGTTTATCCCCCACTTTCAGGTAACTTAGCTCATGCT
GGTGGGTCTGTTGACTTGGCTATCTTTTCTTTACATTTAGCAGGTGTTTCTTCAATTTTAGG
TGCTGTAAATTTTATTACTACAATTATTAATATGCGATGACAAGGTATGCAGTTTGAACGA
TTACCATTATTTGTTTGATCTGTGAAAATTACTGCAATTTTGTTGTTGCTTTCTCTTCCTGTA
CTAGCAGGTGCAATTACTATGCTACTAACTGATCGAAACTTTAATACTTCGTTTTTTGATCC
AGCAGGAGGGGGTGACCCAATTCAGTGATTGCTTTTTAACGGCCGCGGTACCCTGACCGT
GCAAAGGTAGCATAATCATTTGCCTTTTAATTGAAGGCTGGTATGAACGGTTTGACGAGA
GTAAAGCTGTCTCACATATACTTAATAAAAATTAATTTAAAGGTGCAAAAGCCTTTATTAC
CCAAAAAGACGAGAAGACCCTGTTGAGCTTTAATTTAACTCAAGTACTAACTATATATTA
ATATAATCCACTTGCTTGATAAAAATTTTAATTGGGGCGATTAAGGAACACTCAAAAGCTT
CCTGAATACT--
ATGATAAATATTCCATAGATTAGATCGATCCAGCAATGCTGATCAACAAAATGAGTTACC
ACAGGGATAACAGCATAATTCTTTTTGAGAGCTCATATCGAAAAAAGAGATTGTGACCTC
GATGTTGGACTAGGGTAACCGGAAAGGTGCAGCCGC-TTTCCCGTT-TG-
GTCTGTTCGACCAATAATTCCCT
>Nerita_fulgurans
ACTTTATACATTATGTTTGGTGTGTGATCTGGTTTAGTTGGGACTGCTTTGAGACTTTTAAT
TCGAGCTGAACTTGGTCAGCCAGGGGCTCTTTTAGGTGATGATCAGCTTTATAACGTAATT
GTCACTGCTCATGCTTTTGTAATAATTTTCTTTTTGGTAATGCCTATGATGATTGGGGGATT
TGGTAATTGATTGGTTCCTTTAATGTTAGGAGCTCCTGATATGGCGTTTCCTCGATTAAATA
ATATAAGTTTTTGATTACTTCCGCCTTCATTGACTTTATTACTTGCTTCTTCTGCTGTTGAAA
GTGGGGTGGGGACAGGTTGAACAGTATATCCTCCTTTATCTGGGAATTTGGCTCATGCGGG
AGGTTCTGTAGACTTAGCTATTTTTTCATTACACTTAGCTGGTGTTTCTTCAATTTTGGGTG
CTGTAAATTTTATTACCACAATTATTAATATGCGATGACAAGGGTTGCAATTTGAGCGATT
ACCTCTTTTTGTGTGGTCTGTAAAGATTACTGCTATTCTTCTTTTATTATCTTTGCCTGTTCT
TGCAGGTGCGATTACTATGTTGTTAACTGATCGAAATTTTAATACATCTTTCTTTGATCCTG
CCGGAGGTGGAGATCCTATTCAGTGATCAC--
TTTAACGGCCGCGGTACCCTGACCGTGCAAAGGTAGCATAATCATTTGCCTTTTAATTGGA
GGCTAGTATGAATGGTCTGACGAGAGTAAAACTGTCTCACATATATTCAATAGAAATTAA
CCTAAAGGTGCAAAGGCCTTTATCTAATAAAAAGACGAGAAGACCCTGTTGAGCTTTAAT
CTTAA-
TGAGTATTTTTTTTATATTTATAAATTTACTCTACTCACTAAGGATTTTAATTGGGGCGATT
AAGGAACAAGCACAAGCTTCCTGAATTTA--ACTAAAAATGTT-
CATAAATTAAATCGATCCAGCAATGTTGATCAACAAAATGAGTTACCACAGGGATAACAG

CATAATTCTCCTTGAGAGCCCATATCGAAAGGAGAGATTGTGACCTCGATGTTGGACTAG
GGTAACTAG-AAGGTGCAGCCGC-TTTCTTGTT-TG-GTCTGTTCGACCAGTAATTCCCT
>Nerita_tessellata
ACTTTATACATTATGTTTGGTGTGTGATCTGGTTTAGTTGGGACTGCTTTGAGACTTTTAAT
TCGAGCTGAACTTGGTCAGCCAGGGGCTCTTTTAGGTGATGATCAGCTTTATAACGTAATT
GTCACTGCTCATGCTTTTGTAATAATTTTCTTTTTGGTAATGCCTATGATGATTGGGGGATT
TGGTAATTGATTGGTTCCTTTAATGTTAGGAGCTCCTGATATGGCGTTTCCTCGATTAAATA
ATATAAGTTTTTGATTACTTCCGCCTTCATTGACTTTATTACTTGCTTCTTCTGCTGTTGAAA
GTGGGGTGGGGACAGGTTGAACAGTATATCCTCCTTTATCTGGGAATTTGGCTCATGCGGG
AGGTTCTGTAGACTTAGCTATTTTTTCATTACACTTAGCTGGTGTTTCTTCAATTTTGGGTG
CTGTAAATTTTATTACCACAATTATTAATATGCGATGACAAGGGTTGCAATTTGAGCGATT
ACCTCTTTTTGTGTGGTCTGTAAAGATTACTGCTATTCTTCTTTTATTATCTTTGCCTGTTCT
TGCAGGTGCGATTACTATGTTGTTAACTGATCGAAATTTTAATACATCTTTCTTTGATCCTG
CCGGAGGTGGAGATCCTATTCAGTGATCAC--
TTTAACGGCCGCGGTACCCTGACCGTGCAAAGGTAGCATAATCATTTGCCTTTTAATTGGA
GGCTAGTATGAATGGTCTGACGAGAGTAAAACTGTCTCACATATATTCAATAGAAATTAA
CCTAAAGGTGCAAAGGCCTTTATCTAATAAAAAGACGAGAAGACCCTGTTGAGCTTTAAT
CTTAA-
TGAGTATTTTTTTATATTTATAAATTTACTCTACTCACTAAGGATTTTAATTGGGGCGATT
AAGGAACAAGCACAAGCTTCCTGAATTTA--ACTAAAAATGTT-
CATAAATTAAATCGATCCAGCAATGTTGATCAACAAAATGAGTTACCACAGGGATAACAG
CATAATTCTCCTTGAGAGCCCATATCGAAAGGAGAGATTGTGACCTCGATGTTGGACTAG
GGTAACTAG-AAGGTGCAGCCGC-TTTCTTGTTCTG-GTCTGTTCGACCAGTAATTCCCT
>Nerita_peloronta
ACTCTATATATTATGTTTGGTGTGTGATCCGGTTTGGTCGGGACTGCTCTGAGACTTCTGAT
TCGAGCTGAGCTCGGGCAGCCGGGGGCCCTTTTAGGTGATGATCAGCTTTATAATGTAATT
GTAACTGCTCATGCATTTGTAATAATTTTCTTTTTAGTAATGCCTATGATAATTGGAGGATT
TGGTAACTGATTGGTTCCTTTAATGTTGGGTGCTCCTGATATGGCGTTTCCTCGGTTGAATA
ATATAAGTTTTTGGTTGCTTCCTCCTTCATTAACTCTATTGTTGGCTTCTTCTGCTGTCGAG
AGCGGTGTGGGTACAGGTTGGACAGTTTATCCGCCTTTATCTGGTAATCTGGCTCACGCAG
GAGGATCTGTAGACTTGGCCATTTTCTCTTTGCATCTGGCTGGTGTATCTTCGATTTTGGGT
GCTGTAAACTTTATTACTACGATCATTAATATGCGATGGCAGGGAATGCAGTTCGAACGAT
TACCTCTTTTTGTATGGTCAGTTAAGATTACTGCCATTCTTTTGTTATTATCCTTGCCTGTTC
TTGCTGGTGCAATTACTATGTTGTTGACCGATCGAAATTTTAATACATCTTTCTTTGACCCT
GCAGGGGGTGGTGATCCTATCCAGTGATCAC--
TTTAACGGCCGCGGTACCCTGACCGTGCAAAGGTAGCATAATCATTTGCCTTTTAATTGGA
GGCTAGTATGAATGGTCTAACGAGAGTAACACTGTCTCATATATATTCAATAGAAATTAA
CTTAAAGGTGCAAAGGCCTTTATCCATTAAAAAGACGAGAAGACCCTGTTGAGCTTTAAT
CTTAA-
TGAACATTCCTCCTATATTCATAAATTTACCTTGTTCACTAAAGATTTTAATTGGGGCGATT
AAGGAACAAACACTAGCTTCCTGAAATAA--CTCAGAAATATT-
TATAAATTAAACCGATCCAACAATGTTGATCAGCAAAATGAGTTACCACAGGGATAACAG
CATAATTCTTCTTGAGAGTCCCTATCGAAAGAAGAGATTGTGACCTCGATGTTGGACTAGG
GTAACCAG-AAGGTGCAGCCGC-TTTCTTGTT-TG-GTCTGTTCGACCAATAATTCCCT
>Nerita_versicolor
ACTTTATATATTATGTTTGGTGTGTGATCTGGTCTAGTCGGGACTGCCTTAAGACTTTTGAT
TCGGGCTGAGCTCGGACAGCCAGGTGCCCTTTTAGGGGATGATCAGCTTTATAATGTAATT
GTCACTGCGCACGCGTTCGTGATAATTTTCTTTTTAGTGATGCCTATGATAATTGGGGGGT
TTGGTAACTGATTGGTTCCTTTAATGTTGGGTGCTCCCGATATGGCGTTCCCTCGGCTAAAT
AATATAAGTTTCTGACTGCTTCCTCCTTCATTAACTTTACTACTTGCTTCTTCTGCTGTTGA
GAGTGGTGTGGGGACAGGGTGGACAGTTTACCCACCTTTGTCTGGTAATCTGGCTCATGCT
GGAGGGTCTGTAGATTTGGCTATTTTTTCACTACATTTGGCTGGTGTGTCTTCAATTCTAGG
TGCTGTAAATTTTATTACTACGATTATTAATATGCGGTGACAAGGGATGCAGTTTGAGCGG
TTGCCTCTCTTTGTGTGGTCAGTTAAGATTACTGCCATTCTCTTGTTGCTATCTTTACCTGTC
CTTGCTGGTGCGATCACTATGTTGCTGACTGATCGAAACTTTAATACATCCTTTTTTGACCC
GGCAGGAGGTGGAGATCCTATTCAGTGATCAC--
TTTAACGGCCGCGGTACCCTGACCGTGCAAAGGTAGCATAATCATTTGCCTTTTAATTGAA
GGCTAGTATGAATGGTCCGACGAGAGTAAAACTGTCTCATATGTATTCAATAGAAATTAA
CCTAAAGGTGCAAAGGCCTTTATTCACCAAAAAGACGAGAAGACCCTGTTGAGCTTTAAT
CTTAA-

TGAACATTCTTTCTATATTTATAAATCCACTCTGCTCACTAAAGATTTTAATTGGGGCGATT
AAGGAACAAAGACCAGCTTCCTGA-----------------T-
CATAAATTAAACCGATCCAGTAATGCTGATCAACAAAATGAGTTACCACAGGGATAACAG
CATAATTCTTCTTGAGAGTTCCTATCGAAAGAAGAGATTGTGACCTCGATGTTGGACTAGG
GTAACTAG-AAGGTGTAGCCGC-TTTCTTGTT-TG-GTCTGTTCGACCAGTAATTCCCT
>Bathynerita_naticoidea
CTTTGTATCTTTTTGTTTGCTGTATGGGCAGGTTTAGTTGGAACTGCTCTTAGTTTGTTAAT
TCGAGCAGAGCTTGGACAGCCTGGTGCTCTATTAGGTGATGACCAGCTTTATAATGTAATT
GTAACTGCTCATGGTATAATTATGATTTTCTTTTTAGTAATGCCTATGATGATGGGTGGGTT
TGGAAATTGATTAGTTCCACTTATGTTAGGTGCTCCTGACATGGCTTTCCCTCGATTAAAT
AACATAGGTTTTTGACTTCTTCCCCCCGCTTTAACTTTGTTATTAGCTTCTTCAGCTGTAGA
AAGTGGGTCTGGTACAGGATGAACAGTTTATCCTCCATTGGCAGGAAATCTTGCTCATGCA
GGTGGGGCTGTTGATTTAACAATTTTTTCGCTTCATTTAGCTGGAATTTCATCAATTTTGAG
TTCTGTAAATTTTATTACAACAGTAATTAATATGCGATGACAAGGAATGCAGTTCGAGCGA
TTACCTTTGTTTGTGTGATCTGTAAAGATTACTGCAATTATGTTATTACTTTCTTTGCCTGT
GTTAGCTGGTGCAATCACAATGTTGTTAACAGATCGAAATTTTAATACATCTTTCTTTGAC
CCGGCAGGTGGGGGTGATCCAGTGCAGTGATTAC--
TTTAACGGCCGCGGTACCCTGACCGTGCAAAGGTAGCATAATCATTTGCCTTTTAATTGAA
GGCTAGTATGAATGGTTTAACGAGAGTAAAACTGTCTCACATGTATTTTACAGAAATTAAT
TTGAAGGTGCAAAAGCCTTCATTAACTAAAAAGACGAGAAGACCCTGTTGAGCTTTAATT
TTAACTAGATA-
TGCTTTTATATTTATATAATTTATTAATCTAGTAAAAATTTTAATTGGGGCGATTAAGGAA
CAACAAAAAGCTTCCTGAGTTAT--TAACTAAATATT-
GATAAATTAGATCGATCCAGCCATGCTGATCAACAAAATGAGTTACCACAGGGATAACAG
CATAATTCTTTTTAAGAGCTCGTATCGAAAAAAGAGATTGTGACCTCGATGTTGGACTAGG
GTGACAAA-AAGGTGTAGCCGC-TTTTTTGCT-TG-GTCTGTTCGACCAATAATTCCCT

Alignment used to statistical parsimony network analysis.
>JX646671
TTCCTCCATCATTAACTTTGCTATTAGCTTCTTCTGCCGTTGAAAGTGGGGTAGGGACTGGT
TGAACTGTTTATCCTCCGCTTTCAGGTAATTTAGCTCATGCGGGTGGGTCTGTTGACTTAGC
AATTTTTTCTTTACATTTAGCAGGTGTTTCTTCAATTTTAGGTGCTGTGAATTTTATTACCA
CAATTATTAATATGCGATGACAGGGTATGCAATTTGAACGATTACCATTATTTGTTTGATC
TGTAAAAATTACTGCAATTCTGTTGTTGCTTTCTCTTCCTGTGCTAGCAGGTGCAATTACTA
TGCTGTTAACTGATCGAAACTTCAACACTTCATTCTTT
>JF811000
TTCCTCCATCATTAACTTTGCTATTAGCTTCTTCTGCCGTTGAAAGTGGGGTAGGGACTGGT
TGAACTGTTTATCCTCCGCTTTCAGGTAATTTAGCTCATGCGGGTGGGTCTGTTGACTTAGC
AATTTTTTCTTTACATTTAGCAGGTGTTTCTTCAATTTTAGGTGCTGTGAATTTTATTACCA
CAATTATTAATATGCGATGACAGGGTATGCAATTTGAACGATTACCATTATTTGTTTGATC
TGTAAAAATTACTGCAATTCTGTTGTTGCTTTCTCTTCCTGTGCTAGCAGGTGCAATTACTA
TGCTGTTAACTGATCGAAACTTCAACACTTCATTCTTT
>FJ348969
TTCCTCCATCATTAACTTTGCTATTAGCTTCTTCTGCCGTTGAAAGTGGGGTAGGGACTGGT
TGAACTGTTTATCCTCCGCTTTCAGGTAATTTAGCTCATGCGGGTGGGTCTGTTGACTTAGC
AATTTTTTCTTTACATTTAGCAGGTGTTTCTTCAATTTTAGGTGCTGTGAATTTTATTACCA
CAATTATTAATATGCGATGACAGGGTATGCAATTTGAACGATTACCATTATTTGTTTGATC
TGTAAAAATTACTGCAATTCTGTTGTTGCTTTCTCTTCCTGTGCTAGCAGGTGCAATTACTA
TGCTGTTAACTGATCGAAACTTCAACACTTCATTCTTT
>FJ348965
TTCCTCCATCATTAACTTTGCTATTAGCTTCTTCTGCCGTTGAAAGTGGGGTAGGGACTGGT
TGAACTGTTTATCCTCCGCTTTCAGGTAATTTAGCTCATGCGGGTGGGTCTGTTGACTTAGC
AATTTTTTCTTTACATTTAGCAGGTGTTTCTTCAATTTTAGGTGCTGTGAATTTTATTACCA
CAATTATTAATATGCGATGACAGGGTATGCAATTTGAACGATTACCATTATTTGTTTGATC
TGTAAAAATTACTGCAATTCTGTTGTTGCTTTCTCTTCCTGTGCTAGCAGGTGCAATTACTA
TGCTGTTAACTGATCGAAACTTCAACACTTCATTCTTT
>FJ348963
TTCCTCCATCATTAACTTTGCTATTAGCTTCTTCTGCCGTTGAAAGTGGGGTAGGGACTGGT
TGAACTGTTTATCCTCCGCTTTCAGGTAATTTAGCTCATGCGGGTGGGTCTGTTGACTTAGC
AATTTTTTCTTTACATTTAGCAGGTGTTTCTTCAATTTTAGGTGCTGTGAATTTTATTACCA

CAATTATTAATATGCGATGACAGGGTATGCAATTTGAACGATTACCATTATTTGTTTGATC
TGTAAAAATTACTGCAATTCTGTTGTTGCTTTCTCTTCCTGTGCTAGCAGGTGCAATTACTA
TGCTGTTAACTGATCGAAACTTCAACACTTCATTCTTT
>FJ348962
TTCCTCCATCATTAACTTTGCTATTAGCTTCTTCTGCCGTTGAAAGTGGGGTAGGGACTGGT
TGAACTGTTTATCCTCCGCTTTCAGGTAATTTAGCTCATGCGGGTGGGTCTGTTGACTTAGC
AATTTTTTCTTTACATTTAGCAGGTGTTTCTTCAATTTTAGGTGCTGTGAATTTTATTACCA
CAATTATTAATATGCGATGACAGGGTATGCAATTTGAACGATTACCATTATTTGTTTGATC
TGTAAAAATTACTGCAATTCTGTTGTTGCTTTCTCTTCCTGTGCTAGCAGGTGCAATTACTA
TGCTGTTAACTGATCGAAACTTCAACACTTCATTCTTT
>FJ348961
TTCCTCCATCATTAACTTTGCTATTAGCTTCTTCTGCCGTTGAAAGTGGGGTAGGGACTGGT
TGAACTGTTTATCCTCCGCTTTCAGGTAATTTAGCTCATGCGGGTGGGTCTGTTGACTTAGC
AATTTTTTCTTTACATTTAGCAGGTGTTTCTTCAATTTTAGGTGCTGTGAATTTTATTACCA
CAATTATTAATATGCGATGACAGGGTATGCAATTTGAACGATTACCATTATTTGTTTGATC
TGTAAAAATTACTGCAATTCTGTTGTTGCTTTCTCTTCCTGTGCTAGCAGGTGCAATTACTA
TGCTGTTAACTGATCGAAACTTCAACACTTCATTCTTT
>FJ348960
TTCCTCCATCATTAACTTTGCTATTAGCTTCTTCTGCCGTTGAAAGTGGGGTAGGGACTGGT
TGAACTGTTTATCCTCCGCTTTCAGGTAATTTAGCTCATGCGGGTGGGTCTGTTGACTTAGC
AATTTTTTCTTTACATTTAGCAGGTGTTTCTTCAATTTTAGGTGCTGTGAATTTTATTACCA
CAATTATTAATATGCGATGACAGGGTATGCAATTTGAACGATTACCATTATTTGTTTGATC
TGTAAAAATTACTGCAATTCTGTTGTTGCTTTCTCTTCCTGTGCTAGCAGGTGCAATTACTA
TGCTGTTAACTGATCGAAACTTCAACACTTCATTCTTT
>JF810998
TTCCTCCATCATTAACTTTGCTATTAGCTTCTTCTGCCGTTGAAAGTGGGGTAGGGACTGGT
TGAACTGTTTATCCTCCGCTTTCAGGTAATTTAGCTCATGCGGGTGGGTCTGTTGACTTAGC
AATTTTTTCTTTACATTTAGCAGGTGTTTCTTCAATTTTAGGTGCTGTGAATTTTATTACCA
CAATTATTAATATGCGATGACAGGGTATGCAATTTGAACGATTACCATTATTTGTTTGATC
TGTAAAAATTACTGCAATTCTGTTATTGCTTTCTCTTCCTGTGCTAGCAGGTGCAATTACTA
TGCTGTTAACTGATCGAAACTTCAACACTTCATTCTTT
>FJ348973
TTCCTCCATCATTAACTTTGCTATTAGCTTCTTCTGCCGTTGAAAGTGGGGTAGGGACTGGT
TGAACTGTTTATCCTCCGCTTTCAGGTAATTTAGCTCATGCGGGTGGGTCTGTTGACTTAGC
AATTTTTTCTTTACATTTAGCAGGTGTTTCTTCAATTTTAGGTGCTGTGAATTTTATTACCA
CAATTATTAATATGCGATGACAGGGTATGCAATTTGAACGATTACCATTATTTGTTTGATC
TGTAAAAATTACTGCAATTCTGTTGTTGCTTTCTCTTCCTGTGCTGGCAGGTGCAATTACTA
TGCTGTTAACTGATCGAAACTTCAACACTTCATTCTTT
>FJ348968
TTCCTCCATCATTAACTTTGCTATTAGCTTCTTCTGCCGTTGAAAGTGGGGTAGGGACTGGT
TGAACTGTCTATCCTCCGCTTTCAGGTAATTTAGCTCATGCGGGTGGGTCTGTTGACTTAG
CAATTTTTTCTTTACATTTAGCAGGTGTTTCTTCAATTTTAGGTGCTGTGAATTTTATTACC
ACAATTATTAATATGCGATGACAGGGTATGCAATTTGAACGATTACCATTATTTGTTTGAT
CTGTAAAAATTACTGCAATTCTGTTGTTGCTTTCTCTTCCTGTGCTAGCAGGTGCAATTACT
ATGCTGTTAACTGATCGAAACTTCAACACTTCATTCTTT
>FJ348967
TTCCTCCATCATTAACTTTGCTATTAGCTTCTTCTGCCGTTGAAAGTGGGGTAGGAACTGGT
TGAACTGTTTATCCTCCGCTTTCAGGTAATTTAGCTCATGCGGGTGGGTCTGTTGACTTAGC
AATTTTTTCTTTACATTTAGCAGGTGTTTCTTCAATTTTAGGTGCTGTGAATTTTATTACCA
CAATTATTAATATGCGATGACAGGGTATGCAATTTGAACGATTACCATTATTTGTTTGATC
TGTAAAAATTACTGCAATTCTGTTGTTGCTTTCTCTTCCTGTGCTAGCAGGTGCAATTACTA
TGCTGTTAACTGATCGAAACTTCAACACTTCATTCTTT
>FJ348966
TTCCTCCATCATTAACTTTGCTATTAGCTTCTTCTGCCGTTGAAAGTGGGGTAGGGACTGGT
TGAACTGTTTATCCTCCGCTTTCAGGTAATTTAGCTCATGCGGGTGGGTCTGTTGACTTAGC
AATTTTTTCTTTACATTTAGCAGGTGTTTCTTCAATTTTAGGTGCTGTGAATTTTATTACCA
CAATTATTAATATGCGATGACAGGGTATGCAATTTGAACGATTACCATTATTTGTTTGATC
TGTAAAAATTACTGCAATTCTGTTGCTGCTTTCTCTTCCTGTGCTAGCAGGTGCAATTACTA
TGCTGTTAACTGATCGAAACTTCAACACTTCATTCTTT
>MK628554

TTCCTCCATCATTAACTCTGCTATTAGCTTCTTCTGCCGTTGAAAGTGGGGTAGGGACTGG
TTGAACTGTTTATCCTCCGCTTTCAGGTAATTTAGCTCATGCGGGTGGGTCTGTTGACTTAG
CAATTTTTTCTTTACATTTAGCAGGTGTTTCTTCAATTTTAGGTGCTGTGAATTTTATTACC
ACAATTATTAATATGCGATGACAGGGTATGCAATTTGAACGATTACCATTATTTGTTTGAT
CTGTAAAAATTACTGCAATTCTGTTGTTGCTTTCTCTTCCTGTGCTAGCAGGTGCAATTACT
ATGCTGTTAACTGATCGAAACTTCAACACTTCATTCTTT
>MK628552
TTCCTCCATCATTAACTTTGCTATTAGCTTCTTCTGCCGTTGAAAGTGGGGTAGGGACTGGT
TGAACTGTTTATCCTCCGCTTTCAGGTAATTTAGCTCATGCGGGTGGGTCTGTTGACTTAGC
AATTTTTTCTTTACATTTAGCAGGTGTTTCTTCAATTTTAGGTGCTGTGAATTTTATTACCA
CAATTATTAATATGCGATGACAGGGTATGCAATTTGAACGATTACCATTATTTGTTTGATC
TGTAAAAATTACTGCAATTCTGTTGTTGCTTTCTCTTCCTGTGCTAGCAGGTGCAATTACTA
TGCTGTTAACTGATCGAAACTTCAATACTTCATTCTTT
>FJ348964
TTCCTCCATCATTAACTTTGCTATTAGCTTCTTCTGCCGTTGAAAGTGGGGTAGGGACTGGT
TGAACTGTTTATCCTCCGCTTTCAGGTAATTTAGCTCATGCGGGTGGGTCTGTTGACTTAGC
AATTTTTTCTTTACATTTAGCAGGTGTTTCTTCAATTTTAGGTGCTGTGAATTTTATTACCA
CAATTATTAATATGCGATGACAGGGTATGCAATTTGAACGATTACCATTATTTGTTTGATC
TGTAAAAATTACTGCAATTCTGTTGTTGCTTTCTCTTCCTGTGCTAGCAGGTGCAATTACTA
TGCTGTTAACTGATCGAAACTTCAATACTTCATTCTTT
>MK628555
TTCCACCATCATTAACTTTGCTATTAGCTTCTTCTGCCGTTGAAAGTGGGGTAGGGACTGG
TTGAACTGTTTATCCTCCGCTTTCAGGTAATTTAGCTCATGCGGGTGGGTCTGTTGACTTAG
CAATTTTTTCTTTACATTTAGCAGGTGTTTCTTCAATTTTAGGTGCTGTGAATTTTATTACC
ACAATTATTAATATGCGATGACAGGGTATGCAATTTGAACGATTACCATTATTTGTTTGAT
CTGTAAAAATTACTGCAATTCTGTTGCTGCTTTCTCTTCCTGTGCTAGCAGGTGCAATTACT
ATGCTGTTAACTGATCGAAACTTCAACACTTCATTCTTT
>MK628553
TCCCTCCATCATTAACTTTGCTATTAGCTTCTTCTGCCGTTGAAAGTGGGGTAGGAACTGG
TTGAACTGTTTATCCTCCGCTTTCAGGTAATTTAGCTCATGCGGGTGGGTCTGTTGACTTAG
CAATTTTTTCTTTACATTTAGCAGGTGTTTCTTCAATTTTAGGTGCTGTGAATTTTATTACC
ACAATTATTAATATGCGATGACAGGGTATGCAATTTGAACGATTACCATTATTTGTTTGAT
CTGTAAAAATTACTGCAATTCTGTTGTTGCTTTCTCTTCCTGTGCTAGCAGGTGCAATTACT
ATGCTGTTAACTGATCGAAACTTCAACACTTCATTTTTT
>JF811001b
TTCCTCCATCATTAACTTTGCTATTAGCTTCTTCTGCCGTTGAAAGTGGGGTAGGGACTGGT
TGAACTGTTTATCCTCCGCTTTCAGGTAATTTAGCTCATGCGGGTGGGTCTGTTGACTTAGC
AATTTTTTCTTTGCATTTAGCAGGTGTTTCTTCAATTTTAGGTGCTGTGAATTTCATTACCA
CAATTATTAATATGCGATGACAGGGTATGCAATTTGAACGATTACCATTATTTGTTTGATC
TGTAAAAATTACTGCAATTCTGTTGTTGCTTTCTCTTCCTGTGCTAGCAGGTGCAATTACTA
TGCTGTTAACTGATCGAAACTTCAACACTTCATTCTTT
>JF811001a
TTCCTCCATCATTAACTTTGCTATTAGCTTCTTCTGCCGTTGAAAGTGGGGTAGGGACTGGT
TGAACTGTTTATCCTCCGCTTTCAGGTAATTTAGCTCATGCGGGTGGGTCTGTTGACTTAGC
AATTTTTTCTTTGCATTTAGCAGGTGTTTCTTCAATTTTAGGTGCTGTGAATTTCATTACCA
CAATTATTAATATGCGATGACAGGGTATGCAATTTGAACGATTACCATTATTTGTTTGATC
TGTAAAAATTACTGCAATTCTGTTGTTGCTTTCTCTTCCTGTGCTAGCAGGTGCAATTACTA
TGCTGTTAACTGATCGAAACTTCAACACTTCATTCTTT
>FJ348957
TTCCTCCATCATTGACTTTGCTATTAGCTTCTTCTGCCGTTGAAAGTGGGGTAGGGACTGGT
TGAACTGTTTATCCTCCGCTTTCAGGTAATTTAGCTCATGCGGGTGGGTCTGTTGACTTAGC
AATTTTTTCTTTGCATTTAGCAGGTGTTTCTTCAATTTTAGGTGCTGTGAATTTTATTACCA
CAATTATTAATATGCGATGACAGGGTATGCAATTTGAACGATTACCATTATTTGTTTGATC
TGTAAAAATTACTGCAATTCTGTTGTTGCTTTCTCTTCCTGTGCTAGCAGGTGCAATTACTA
TGCTGTTAACTGATCGAAACTTCAACACTTCATTCTTT
>FJ348956
TTCCTCCATCATTGACTTTGCTATTAGCTTCTTCTGCCGTTGAAAGTGGGGTAGGGACTGGT
TGAACTGTTTATCCTCCGCTTTCAGGTAATTTAGCTCATGCGGGTGGGTCTGTTGACTTAGC
AATTTTTTCTTTGCATTTAGCAGGTGTTTCTTCAATTTTAGGTGCTGTGAATTTTATTACCA
CAATTATTAATATGCGATGACAGGGTATGCAATTTGAACGATTACCATTATTTGTTTGATC

TGTAAAAATCACTGCAATTCTGTTGTTGCTTTCTCTTCCTGTGCTAGCAGGTGCAATTACTA
TGCTGTTAACTGATCGAAACTTCAACACTTCATTCTTT
>JX646668
TTCCTCCATCACTAACTTTGCTATTAGCTTCTTCTGCCGTTGAAAGTGGGGTAGGGACTGG
TTGAACTGTTTATCCTCCGCTTTCAGGTAATTTAGCTCATGCGGGTGGGTCTGTTGACTTAG
CAATTTTTTCTTTGCATTTAGCAGGTGTTTCTTCAATTTTAGGTGCTGTGAATTTTATCACC
ACAATTATTAATATGCGATGACAGGGTATGCAATTTGAACGATTACCATTATTTGTTTGAT
CTGTAAAAATTACTGCAATTCTGTTGTTGCTTTCTCTTCCTGTGCTAGCAGGTGCAATTACT
ATGCTGTTAACTGATCGAAACTTCAACACTTCATTCTTT
>FJ348970
TTCCTCCATCACTAACTTTGCTATTAGCTTCTTCTGCCGTTGAAAGTGGGGTAGGGACTGG
TTGAACTGTTTATCCTCCGCTTTCAGGTAATTTAGCTCATGCGGGTGGGTCTGTTGACTTAG
CAATTTTTTCTTTGCATTTAGCAGGTGTTTCTTCAATTTTAGGTGCTGTGAATTTTATTACC
ACAATTATTAATATGCGATGACAGGGTATGCAATTTGAACGATTACCATTATTTGTTTGAT
CTGTAAAAATTACTGCAATTCTGTTGTTGCTTTCTCTTCCTGTGCTAGCAGGTGCAATTACT
ATGCTGTTAACTGATCGAAACTTCAATACTTCATTCTTT
>FJ348959
TTCCTCCATCATTAACTTTGCTATTAGCTTCTTCTGCCGTTGAAAGTGGGGTAGGGACTGGT
TGAACTGTTTATCCTCCGCTTTCAGGTAATTTGGCTCATGCGGGCGGGTCTGTTGACTTAG
CAATTTTTTCTTTGCATTTAGCAGGTGTTTCTTCAATTTTAGGTGCTGTGAATTTTATTACC
ACAATTATTAATATGCGATGACAGGGTATGCAATTTGAACGATTACCATTATTTGTTTGAT
CTGTAAAAATTACTGCAATTCTGTTGTTGCTTTCTCTTCCTGTGCTAGCAGGTGCAATTACT
ATGCTGTTAACTGATCGAAACTTCAACACTTCATTCTTT
>FJ348958
TTCCTCCATCATTAACTTTGCTATTAGCTTCTTCTGCCGTTGAAAGTGGGGTAGGGACTGGT
TGAACTGTTTATCCTCCGCTTTCAGGTAATTTGGCTCATGCGGGTGGGTCTGTTGACTTAGC
AATTTTTTCTTTGCATTTAGCAGGTGTTTCTTCAATTTTAGGTGCTGTGAATTTTATTACCA
CAATTATTAATATGCGATGACAGGGTATGCAATTTGAACGATTACCATTATTCGTTTGATC
TGTAAAAATTACTGCAATTCTGTTGTTGCTTTCTCTTCCTGTGCTAGCAGGTGCAATTACTA
TACTGTTAACTGATCGAAACTTCAACACTTCATTCTTT
>JF811004
TTCCTCCATCATTAACTTTGCTATTAGCTTCTTCTGCCGTTGAAAGCGGGGTAGGGACTGG
TTGAACTGTTTATCCTCCACTTTCAGGTAATTTAGCTCATGCGGGTGGGTCTGTTGACTTAG
CAATTTTTTCTTTGCATTTAGCAGGTGTTTCTTCAATTTTAGGTGCTGTGAATTTTATTACC
ACAATTATTAATATGCGATGACAGGGTATGCAATTTGAACGATTACCATTATTTGTTTGAT
CTGTAAAAATTACTGCAATCCTGTTGTTGCTTTCTCTTCCTGTGCTAGCAGGTGCAATTACT
ATGCTGTTAACTGATCGAAACTTCAATACTTCATTCTTC
>FJ348975
TTCCTCCATCATTAACTTTGCTATTAGCTTCTTCTGCCGTTGAAAGCGGGGTAGGGACTGG
TTGAACTGTTTATCCTCCACTTTCAGGTAATTTAGCTCATGCGGGTGGGTCTGTTGACTTAG
CAATTTTTTCTTTGCATTTAGCAGGTGTTTCTTCAATTTTAGGTGCTGTGAATTTTATTACC
ACAATTATTAATATGCGATGACAGGGTATGCAATTTGAACGATTACCATTATTTGTTTGAT
CTGTAAAAATTACTGCAATCCTGTTGTTGCTTTCTCTTCCTGTGCTAGCAGGTGCAATTACT
ATGCTGTTAACTGATCGAAACTTCAATACTTCATTCTTC
>JF811003
TTCCTCCATCATTAACTTTGCTATTAGCTTCTTCTGCCGTTGAAAGCGGGGTAGGGACTGG
TTGAACTGTTTATCCTCCACTTTCAGGTAATTTAGCTCATGCGGGTGGGTCTGTTGACTTAG
CAATTTTTTCTTTGCATTTAGCGGGTGTTTCTTCAATTTTAGGTGCTGTGAATTTTATTACC
ACAATTATTAATATGCGATGACAGGGTATGCAATTTGAACGATTACCATTATTTGTTTGAT
CTGTAAAAATTACTGCAATTCTGTTGTTGCTTTCTCTTCCTGTGCTAGCAGGTGCAATTACT
ATGCTGTTAACTGATCGAAACTTCAACACTTCATTCTTT
>FJ348954
TTCCTCCATCATTAACTTTGCTATTAGCTTCTTCTGCCGTTGAAAGCGGGGTAGGGACTGG
TTGAACTGTTTATCCTCCACTTTCAGGTAATTTAGCTCATGCGGGTGGGTCTGTTGACTTAG
CAATTTTTTCTTTGCATTTAGCAGGTGTTTCTTCAATTTTAGGTGCTGTGAATTTTATTACC
ACAATTATTAATATGCGATGACAGGGTATGCAATTTGAACGATTACCATTATTTGTTTGAT
CTGTAAAAATTACTGCAATTCTGTTGTTGCTTTCTCTTCCTGTGCTAGCAGGTGCAATTACT
ATGCTGTTAACTGATCGAAACTTCAACACTTCATTCTTT
>FJ348953

TTCCTCCATCATTAACTTTGCTATTAGCTTCTTCTGCCGTTGAAAGCGGGGTAGGGACTGG
TTGAACTGTTTATCCTCCACTTTCAGGTAATTTAGCTCATGCGGGTGGGTCTGTTGACTTAG
CAATTTTTTCTTTGCATTTAGCAGGTGTTTCTTCAATTTTAGGTGCTGTGAATTTTATTACC
ACAATTATTAATATGCGATGACAGGGTATGCAATTTGAACGATTACCATTATTTGTTTGAT
CTGTAAAAATTACTGCAATTCTGTTGTTGCTTTCTCTTCCTGTGCTAGCAGGTGCAATTACT
ATGCTGTTAACTGATCGAAACTTCAACACTTCATTCTTT
>FJ348951
TTCCTCCATCATTAACTTTGCTATTAGCTTCTTCTGCCGTTGAAAGCGGGGTAGGGACTGG
TTGAACTGTTTATCCTCCACTTTCAGGTAATTTAGCTCATGCGGGTGGGTCTGTTGACTTAG
CAATTTTTTCTTTGCATTTAGCAGGTGTTTCTTCAATTTTAGGTGCTGTGAATTTTATTACC
ACAATTATTAATATGCGATGACAGGGTATGCAATTTGAACGATTACCATTATTTGTTTGAT
CTGTAAAAATTACTGCAATTCTGTTGTTGCTTTCTCTTCCTGTGCTAGCAGGTGCAATTACT
ATGCTGTTAACTGATCGAAACTTCAACACTTCATTCTTT
>FJ348950
TTCCTCCATCATTAACTTTGCTATTAGCTTCTTCTGCCGTTGAAAGCGGGGTAGGGACTGG
TTGAACTGTTTATCCTCCACTTTCAGGTAATTTAGCTCATGCGGGTGGGTCTGTTGACTTAG
CAATTTTTTCTTTGCATTTAGCAGGTGTTTCTTCAATTTTAGGTGCTGTGAATTTTATTACC
ACAATTATTAATATGCGATGACAGGGTATGCAATTTGAACGATTACCATTATTTGTTTGAT
CTGTAAAAATTACTGCAATTCTGTTGTTGCTTTCTCTTCCTGTGCTAGCAGGTGCAATTACT
ATGCTGTTAACTGATCGAAACTTCAACACTTCATTCTTT
>FJ348949
TTCCTCCATCATTAACTTTGCTATTAGCTTCTTCTGCCGTTGAAAGCGGGGTAGGGACTGG
TTGAACTGTTTATCCTCCACTTTCAGGTAATTTAGCTCATGCGGGTGGGTCTGTTGACTTAG
CAATTTTTTCTTTGCATTTAGCAGGTGTTTCTTCAATTTTAGGTGCTGTGAATTTTATTACC
ACAATTATTAATATGCGATGACAGGGTATGCAATTTGAACGATTACCATTATTTGTTTGAT
CTGTAAAAATTACTGCAATTCTGTTGTTGCTTTCTCTTCCTGTGCTAGCAGGTGCAATTACT
ATGCTGTTAACTGATCGAAACTTCAACACTTCATTCTTT
>FJ348948
TTCCTCCATCATTAACTTTGCTATTAGCTTCTTCTGCCGTTGAAAGCGGGGTAGGGACTGG
TTGAACTGTTTATCCTCCACTTTCAGGTAATTTAGCTCATGCGGGTGGGTCTGTTGACTTAG
CAATTTTTTCTTTGCATTTAGCAGGTGTTTCTTCAATTTTAGGTGCTGTGAATTTTATTACC
ACAATTATTAATATGCGATGACAGGGTATGCAATTTGAACGATTACCATTATTTGTTTGAT
CTGTAAAAATTACTGCAATTCTGTTGTTGCTTTCTCTTCCTGTGCTAGCAGGTGCAATTACT
ATGCTGTTAACTGATCGAAACTTCAACACTTCATTCTTT
>FJ348955
TTCCTCCATCATTAACTTTGCTATTAGCTTCTTCTGCCGTTGAAAGCGGGGTAGGGACTGG
TTGAACTGTTTATCCTCCACTTTCAGGTAATTTAGCTCATGCGGGTGGGTCTGTTGACTTAG
CAATTTTTTCTTTGCATTTAGCAGGTGTTTCTTCAATTTTAGGTGCTGTGAATTTTATTACC
ACAATTATTAATATGCGATGACAGGGTATGCAATTTGAACGATTACCATTATTTGTTTGAT
CTGTAAAAATTACTGCAATTCTGTTGTTGCTTTCTCTTCCTGTGCTAGCAGGTGCAATTACT
ATGCTGTTAACTGATCGAAACTTCAATACTTCATTCTTT
>FJ348952
TTCCTCCATCATTAACTTTGCTATTAGCTTCTTCTGCCGTTGAAAGCGGGGTAGGGACTGG
TTGAACTGTTTATCCTCCACTTTCAGGTAATTTAGCTCATGCGGGTGGGTCTGTTGACTTAG
CAATTTTTTCTTTGCATTTAGCAGGTGTTTCTTCAATTTTAGGTGCCGTGAATTTTATTACC
ACAATTATTAATATGCGATGACAGGGTATGCAATTTGAACGATTACCATTATTTGTTTGAT
CTGTAAAAATTACTGCAATTCTGTTGTTGCTTTCTCTTCCTGTGCTAGCAGGTGCAATTACT
ATGCTGTTAACTGATCGAAACTTCAACACTTCATTCTTT
>JF811002b
TTCCTCCATCATTAACTTTGCTATTAGCTTCTTCTGCCGTTGAAAGCGGGGTAGGGACTGG
TTGAACTGTTTATCCTCCACTTTCAGGTAATTTAGCTCATGCGGGTGGGTCTGTTGACTTAG
CAATTTTTTCTTTGCATTTAGCAGGTGTTTCTTCAATTTTAGGTGCTGTGAATTTTATTACC
ACAATTATCAATATGCGATGACAGGGTATGCAATTTGAACGATTACCATTATTTGTTTGAT
CTGTAAAAATTACTGCAATTCTGTTGTTGCTTTCTCTTCCTGTGCTAGCAGGTGCAATTACT
ATGCTGTTAACTGATCGAAACTTCAACACTTCATTCTTT
>JF811002a
TTCCTCCATCATTAACTTTGCTATTAGCTTCTTCTGCCGTTGAAAGCGGGGTAGGGACTGG
TTGAACTGTTTATCCTCCACTTTCAGGTAATTTAGCTCATGCGGGTGGGTCTGTTGACTTAG
CAATTTTTTCTTTGCATTTAGCAGGTGTTTCTTCAATTTTAGGTGCTGTGAATTTTATTACC
ACAATTATCAATATGCGATGACAGGGTATGCAATTTGAACGATTACCATTATTTGTTTGAT

CTGTAAAAATTACTGCAATTCTGTTGTTGCTTTCTCTTCCTGTGCTAGCAGGTGCAATTACT
ATGCTGTTAACTGATCGAAACTTCAACACTTCATTCTTT
>FJ348943
TTCCTCCATCATTAACTTTGCTATTAGCTTCTTCTGCCGTTGAAAGCGGGGTAGGGACTGG
TTGAACTGTTTATCCTCCACTTTCAGGTAATTTAGCTCATGCGGGTGGGTCTGTTGACTTAG
CAATTTTTTCTTTGCATTTAGCAGGTGTTTCTTCAATTTTAGGTGCTGTGAATTTTATTACC
ACAATTATCAATATGCGATGACAGGGTATGCAATTTGAACGATTACCATTATTTGTTTGAT
CTGTAAAAATTACTGCAATTCTGTTGTTGCTTTCTCTTCCTGTGCTAGCAGGTGCAATTACT
ATGCTGTTAACTGATCGAAACTTCAACACTTCATTCTTT
>FJ348941
TTCCTCCATCATTAACTTTGCTATTAGCTTCTTCTGCCGTTGAAAGCGGGGTAGGGACTGG
TTGAACTGTTTATCCTCCACTTTCAGGTAATTTAGCTCATGCGGGTGGGTCTGTTGACTTAG
CAATTTTTTCTTTGCATTTAGCAGGTGTTTCTTCAATTTTAGGTGCTGTGAATTTTATTACC
ACAATTATCAATATGCGATGACAGGGTATGCAATTTGAACGATTACCATTATTTGTTTGAT
CTGTAAAAATTACTGCAATTCTGTTGTTGCTTTCTCTTCCTGTGCTAGCAGGTGCAATTACT
ATGCTGTTAACTGATCGAAACTTCAACACTTCATTCTTT
>FJ348940
TTCCTCCATCATTAACTTTGCTATTAGCTTCTTCTGCCGTTGAAAGCGGGGTAGGGACTGG
TTGAACTGTTTATCCTCCACTTTCAGGTAATTTAGCTCATGCGGGTGGGTCTGTTGACTTAG
CAATTTTTTCTTTGCATTTAGCAGGTGTTTCTTCAATTTTAGGTGCTGTGAATTTTATTACC
ACAATTATCAATATGCGATGACAGGGTATGCAATTTGAACGATTACCATTATTTGTTTGAT
CTGTAAAAATTACTGCAATTCTGTTGTTGCTTTCTCTTCCTGTGCTAGCAGGTGCAATTACT
ATGCTGTTAACTGATCGAAACTTCAACACTTCATTCTTT
>FJ348939
TTCCTCCATCATTAACTTTGCTATTAGCTTCTTCTGCCGTTGAAAGCGGGGTAGGGACTGG
TTGAACTGTTTATCCTCCACTTTCAGGTAATTTAGCTCATGCGGGTGGGTCTGTTGACTTAG
CAATTTTTTCTTTGCATTTAGCAGGTGTTTCTTCAATTTTAGGTGCTGTGAATTTTATTACC
ACAATTATCAATATGCGATGACAGGGTATGCAATTTGAACGATTACCATTATTTGTTTGAT
CTGTAAAAATTACTGCAATTCTGTTGTTGCTTTCTCTTCCTGTGCTAGCAGGTGCAATTACT
ATGCTGTTAACTGATCGAAACTTCAACACTTCATTCTTT
>FJ348938
TTCCTCCATCATTAACTTTGCTATTAGCTTCTTCTGCCGTTGAAAGCGGGGTAGGGACTGG
TTGAACTGTTTATCCTCCACTTTCAGGTAATTTAGCTCATGCGGGTGGGTCTGTTGACTTAG
CAATTTTTTCTTTGCATTTAGCAGGTGTTTCTTCAATTTTAGGTGCTGTGAATTTTATTACC
ACAATTATCAATATGCGATGACAGGGTATGCAATTTGAACGATTACCATTATTTGTTTGAT
CTGTAAAAATTACTGCAATTCTGTTGTTGCTTTCTCTTCCTGTGCTAGCAGGTGCAATTACT
ATGCTGTTAACTGATCGAAACTTCAACACTTCATTCTTT
>FJ348937
TTCCTCCATCATTAACTTTGCTATTAGCTTCTTCTGCCGTTGAAAGCGGGGTAGGGACTGG
TTGAACTGTTTATCCTCCACTTTCAGGTAATTTAGCTCATGCGGGTGGGTCTGTTGACTTAG
CAATTTTTTCTTTGCATTTAGCAGGTGTTTCTTCAATTTTAGGTGCTGTGAATTTTATTACC
ACAATTATCAATATGCGATGACAGGGTATGCAATTTGAACGATTACCATTATTTGTTTGAT
CTGTAAAAATTACTGCAATTCTGTTGTTGCTTTCTCTTCCTGTGCTAGCAGGTGCAATTACT
ATGCTGTTAACTGATCGAAACTTCAACACTTCATTCTTT
>FJ348936
TTCCTCCATCATTAACTTTGCTATTAGCTTCTTCTGCCGTTGAAAGCGGGGTAGGGACTGG
TTGAACTGTTTATCCTCCACTTTCAGGTAATTTAGCTCATGCGGGTGGGTCTGTTGACTTAG
CAATTTTTTCTTTGCATTTAGCAGGTGTTTCTTCAATTTTAGGTGCTGTGAATTTTATTACC
ACAATTATCAATATGCGATGACAGGGTATGCAATTTGAACGATTACCATTATTTGTTTGAT
CTGTAAAAATTACTGCAATTCTGTTGTTGCTTTCTCTTCCTGTGCTAGCAGGTGCAATTACT
ATGCTGTTAACTGATCGAAACTTCAACACTTCATTCTTT
>FJ348935
TTCCTCCATCATTAACTTTGCTATTAGCTTCTTCTGCCGTTGAAAGCGGGGTAGGGACTGG
TTGAACTGTTTATCCTCCACTTTCAGGTAATTTAGCTCATGCGGGTGGGTCTGTTGACTTAG
CAATTTTTTCTTTGCATTTAGCAGGTGTTTCTTCAATTTTAGGTGCTGTGAATTTTATTACC
ACAATTATCAATATGCGATGACAGGGTATGCAATTTGAACGATTACCATTATTTGTTTGAT
CTGTAAAAATTACTGCAATTCTGTTGTTGCTTTCTCTTCCTGTGCTAGCAGGTGCAATTACT
ATGCTGTTAACTGATCGAAACTTCAACACTTCATTCTTT
>FJ348934

TTCCTCCATCATTAACTTTGCTATTAGCTTCTTCTGCCGTTGAAAGCGGGGTAGGGACTGG
TTGAACTGTTTATCCTCCACTTTCAGGTAATTTAGCTCATGCGGGTGGGTCTGTTGACTTAG
CAATTTTTTCTTTGCATTTAGCAGGTGTTTCTTCAATTTTAGGTGCTGTGAATTTTATTACC
ACAATTATCAATATGCGATGACAGGGTATGCAATTTGAACGATTACCATTATTTGTTTGAT
CTGTAAAAATTACTGCAATTCTGTTGTTGCTTTCTCTTCCTGTGCTAGCAGGTGCAATTACT
ATGCTGTTAACTGATCGAAACTTCAACACTTCATTCTTT
>FJ348933
TTCCTCCATCATTAACTTTGCTATTAGCTTCTTCTGCCGTTGAAAGCGGGGTAGGGACTGG
TTGAACTGTTTATCCTCCACTTTCAGGTAATTTAGCTCATGCGGGTGGGTCTGTTGACTTAG
CAATTTTTTCTTTGCATTTAGCAGGTGTTTCTTCAATTTTAGGTGCTGTGAATTTTATTACC
ACAATTATCAATATGCGATGACAGGGTATGCAATTTGAACGATTACCATTATTTGTTTGAT
CTGTAAAAATTACTGCAATTCTGTTGTTGCTTTCTCTTCCTGTGCTAGCAGGTGCAATTACT
ATGCTGTTAACTGATCGAAACTTCAACACTTCATTCTTT
>FJ348932
TTCCTCCATCATTAACTTTGCTATTAGCTTCTTCTGCCGTTGAAAGCGGGGTAGGGACTGG
TTGAACTGTTTATCCTCCACTTTCAGGTAATTTAGCTCATGCGGGTGGGTCTGTTGACTTAG
CAATTTTTTCTTTGCATTTAGCAGGTGTTTCTTCAATTTTAGGTGCTGTGAATTTTATTACC
ACAATTATCAATATGCGATGACAGGGTATGCAATTTGAACGATTACCATTATTTGTTTGAT
CTGTAAAAATTACTGCAATTCTGTTGTTGCTTTCTCTTCCTGTGCTAGCAGGTGCAATTACT
ATGCTGTTAACTGATCGAAACTTCAACACTTCATTCTTT
>FJ348945
TTCCTCCATCATTAACTTTGCTATTAGCTTCTTCTGCCGTTGAAAGCGGGGTAGGGACTGG
TTGAACTGTTTATCCTCCACTTTCAGGTAATTTAGCTCATGCGGGTGGGTCTGTTGACTTAG
CAATTTTTTCTTTGCATTTAGCAGGTGTTTCTTCAATTTTAGGTGCTGTGAATTTTATTACC
ACAATTATCAATATGCGATGACAGGGTATGCAATTTGAACGATTACCATTATTTGTTTGAT
CTGTAAAAATTACTGCAATTCTGTTGTTGCTTTCTCTTCCTGTACTAGCAGGTGCAATTACT
ATGCTGTTAACTGATCGAAACTTCAACACTTCATTCTTT
>FJ348942
TTCCTCCATCATTAACTTTGCTATTAGCTTCTTCTGCCGTTGAAAGCGGGGTAGGGACTGG
TTGAACTGTTTATCCTCCACTTTCAGGTAATTTAGCTCATGCGGGCGGGTCTGTTGACTTAG
CAATTTTTTCTTTGCATTTAGCAGGTGTTTCTTCAATTTTAGGTGCTGTGAATTTTATTACC
ACAATTATCAATATGCGATGACAGGGTATGCAATTTGAACGATTACCATTATTTGTTTGAT
CTGTAAAAATTACTGCAATTCTGTTGTTGCTTTCTCTTCCTGTGCTAGCAGGTGCAATTACT
ATGCTGTTAACTGATCGAAACTTCAACACTTCATTCTTT
>FJ348944
TTCCTCCATCATTAACTTTGCTATTAGCTTCTTCTGCCGTCGAAAGCGGGGTAGGGACTGG
TTGAACTGTTTATCCTCCACTTTCAGGTAATTTAGCTCATGCGGGTGGGTCTGTTGACTTAG
CAATTTTTTCTTTGCATTTAGCAGGTGTTTCTTCAATTTTAGGTGCTGTGAATTTTATTACC
ACAATTATTAATATGCGATGACAGGGTATGCAATTTGAACGATTACCATTATTTGTTTGAT
CTGTAAAAATCACTGCAATTCTGTTGTTGCTTTCTCTTCCTGTGCTAGCAGGTGCAATTACT
ATGCTGTTAACTGATCGAAACTTCAACACTTCATTCTTT
>JF810999
TTCCTCCATCATTAACTTTGCTATTAGCTTCTTCTGCCGTCGAAAGCGGGGTAGGGACTGG
TTGAACTGTTTATCCTCCACTTTCAGGTAATTTAGCTCATGCGGGTGGGTCTGTTGACTTGG
CAATTTTTTCTTTGCATTTAGCAGGTGTTTCTTCAATTTTAGGTGCTGTGAATTTTATTACC
ACAATTATCAATATGCGATGACAGGGTATGCAATTTGAACGATTACCATTATTTGTTTGAT
CTGTAAAAATTACTGCAATTCTGTTGTTGCTTTCTCTTCCTGTGCTAGCAGGTGCAATTACT
ATGCTGTTAACTGATCGAAACTTCAACACTTCATTCTTT
>FJ348947
TTCCTCCGTCATTAACTTTGCTATTAGCTTCTTCTGCCGTTGAAAGCGGGGTAGGGACTGG
TTGAACTGTTTATCCTCCACTTTCAGGTAATTTAGCTCATGCGGGCGGGTCTGTTGACTTAG
CAATTTTTTCTTTGCATTTAGCAGGTGTTTCTTCAATTTTAGGTGCTGTGAATTTTATTACC
ACAATTATCAATATGCGATGACAGGGTATGCAATTTGAACGATTACCATTATTTGTTTGAT
CTGTAAAAATTACTGCAATTTTGTTGTTGCTTTCTCTTCCTGTGCTAGCAGGTGCAATTACT
ATGCTGTTAACTGATCGAAACTTCAACACTTCATTCTTT
>FJ977766
TTCCTCCATCATTAACTTTGCTATTAGCTTCTTCTGCCGTTGAAAGCGGGGTAGGGACTGG
TTGAACTGTTTATCCTCCACTTTCAGGTAATTTAGCTCATGCGGGTGGGTCTGTTGACTTAG
CAATTTTTTCTTTGCATTTAGCAGGTGTTTCTTCAATTTTAGGTGCTGTGAATTTTATTACC
ACAATTATTAATATGCGATGACAGGGTATGCAATTTGAACGATTACCATTATTTGTTTGAT

CTGTAAAAATTACTGCAATTTTGTTGTTGCTTTCTCTTCCTGTGCTAGCAGGTGCAATTACT
ATGCTGTTAACTGATCGAAACTTCAACACTTCATTCTTT
>FJ348971
TTCCTCCATCATTAACTTTGCTATTAGCTTCTTCTGCCGTTGAAAGTGGGGTAGGGACTGGT
TGAACTGTTTATCCTCCACTTTCAGGTAATTTAGCTCATGCGGGTGGGTCTGTTGACTTAGC
AATTTTTTCTTTACATCTAGCAGGTGTTTCTTCAATTTTAGGTGCTGTGAATTTTATTACCA
CAATTATTAATATGCGATGACAGGGTATGCAATTTGAACGATTACCATTATTTGTTTGATC
TGTAAAAATTACTGCAATTCTGTTGTTGCTTTCTCTTCCTGTGCTAGCAGGTGCAATTACTA
TGTTGTTAACTGATCGAAACTTCAACACTTCATTCTTT
>FJ348972
TTCCTCCATCATTAACTTTGCTATTAGCCTCTTCTGCCGTTGAAAGTGGGGTAGGGACTGG
TTGAACTGTTTATCCTCCGCTTTCAGGTAATTTAGCTCATGCGGGTGGGTCTGTTGACTTAG
CAATTTTTTCTTTACATTTAGCAGGTGTTTCTTCAATTTTAGGTGCTGTGAATTTTATTACC
ACAATTATTAATATGCGATGACAGGGTATGCAATTTGAACGATTACCGTTATTTGTTTGAT
CTGTAAAAATTACTGCAATTCTGTTGTTGCTTTCTCTTCCTGTGCTAGCAGGTGCAATTACT
ATGCTGTTAACTGATCGAAATTTCAACACTTCATTCTTT
>FJ348946
TTCCCCCATCATTAACTTTGCTATTAGCTTCTTCTGCCGTTGAAAGTGGGGTAGGGACTGG
TTGAACTGTTTATCCTCCACTTTCAGGTAATTTAGCTCATGCGGGTGGGTCTGTTGACTTAG
CAATTTTTTCTTTGCATTTAGCAGGTGTTTCTTCAATTTTAGGTGCTGTGAATTTTATTACC
ACAATTATCAATATGCGATGACAGGGTATGCAATTTGAACGATTACCATTATTTGTTTGAT
CTGTAAAAATTACTGCAATTCTGTTGTTGCTTTCTCTTCCTGTGCTAGCAGGTGCAATTACT
ATGCTATTAACTGATCGAAACTTCAACACTTCATTCTTT
>FJ348974
TTCCTCCATCATTAACTCTGCTATTAGCTTCTTCTGCCGTTGAAAGTGGGGTAGGGACTGG
TTGAACTGTTTATCCTCCGCTTTCAGGTAATTTAGCTCATGCGGGCGGGTCTGTTGACTTAG
CAATTTTTTCTTTGCATTTAGCAGGTGTTTCTTCAATTTTAGGTGCTGTGAATTTTATTACC
ACAATTATTAATATGCGATGACAGGGTATGCAATTTGAACGACTACCATTATTTGTTTGAT
CTGTAAAAATTACTGCAATTCTGTTATTGCTTTCTCTTCCTGTGCTAGCAGGGGCAATTACT
ATGCTGTTAACTGATCGAAACTTCAACACTTCATTCTTT
>MK628548
TCCCTCCATCATTAACTTTATTACTAGCTTCTTCTGCTGTTGAAAGTGGGGTGGGAACTGGT
TGAACTGTTTATCCTCCACTTTCAGGTAATCTTGCTCATGCTGGTGGGTCTGTTGATTTAGC
TATTTTTTCTTTGCATTTAGCGGGTGTTTCCTCAATCCTAGGTGCTGTAAACTTTATTACTA
CAATTATTAACATGCGATGACAGGGGATGCAGTTTGAACGATTGCCATTGTTTGTTTGATC
TGTAAAGATTACTGCAATTTTGTTATTACTTTCTCTTCCTGTGTTAGCAGGGGCAATTACTA
TATTATTGACTGATCGAAACTTTAATACTTCATTTTTT
>MK628550
TCCCTCCATCATTAACTTTATTACTAGCTTCTTCTGCTGTTGAAAGTGGGGTGGGAACTGGT
TGAACTGTTTATCCTCCACTTTCAGGTAATCTTGCTCATGCTGGTGGGTCTGTTGATTTAGC
TATTTTTTCTTTGCATTTAGCGGGTGTTTCCTCAATCCTAGGTGCTGTAAACTTTATTACTA
CAATTATTAACATGCGATGACAGGGGATGCAGTTTGAACGATTGCCATTGTTTGTTTGATC
TGTAAAGATTACTGCAATTTTGTTATTACTTTCTCTTCCTGTGTTAGCAGGGGCAATTACTA
TATTATTGACTGATCGAAACTTTAATACTTCATTTTTT
>MK628549
TCCCTCCATCATTAACTTTATTACTAGCTTCTTCTGCTGTTGAAAGTGGGGTGGGAACTGGT
TGAACTGTTTATCCTCCACTTTCAGGTAATCTTGCTCATGCTGGTGGGTCTGTTGATTTAGC
TATTTTTTCTTTGCATTTAGCGGGTGTTTCCTCAATCCTAGGTGCTGTAAACTTTATTACTA
CAATTATTAACATGCGATGACAGGGGATGCAGTTTGAACGATTGCCATTGTTTGTTTGATC
TGTAAAGATTACTGCAATTTTGTTATTACTTTCTCTTCCTGTGTTAGCAGGGGCAATTACTA
TATTATTGACTGATCGAAACTTTAATACTTCATTTTTT
>MK628551
TCCCTCCATCATTAACTTTATTACTAGCTTCTTCTGCTGTTGAAAGTGGGGTGGGAACTGGT
TGAACTGTTTATCCTCCACTTTCAGGTAATCTTGCTCATGCTGGTGGGTCTGTTGATTTAGC
TATTTTTTCTTTGCATTTAGCGGGTGTTTCCTCAATCCTAGGTGCTGTAAACTTTATTACTA
CAATTATTAACATGCGATGACAGGGGATGCAGTTTGAACGATTGCCATTGTTTGTTTGATC
TGTAAAGATTACTGCAATTTTGTTATTACTTTCTCTTCCTGTGTTAGCAGGGGCAATTACTA
TATTATTGACTGATCGAAACTTTAATACTTCATTTTTT
