# Supplementary material for: Molecular evidences confirm the taxonomic separation of two sympatric congeneric species (Mollusca, Gastropoda, Neritidae, Neritina)
Source: Zookeys. 2020 Jan 16;904:117–30. doi: 10.3897/zookeys.904.46790 (PMC6978406; doi:10.3897/zookeys.904.46790)

Molecular evidences confirm the taxonomic separation of two sympatric congeneric species (Mollusca, Gastropoda, Neritidae, *Neritina*)

Cristiane Xerez Barroso[1,2], João Eduardo Pereira de Freitas[3], Helena Matthews-Cascon[1,2], Luis Ernesto Arruda Bezerra[1,4] & Tito Monteiro da Cruz Lotufo[5]

Supplementary file _2 - Radulae of the *Neritina meleagris* (A) and *Neritina virginea* (B) analysed.

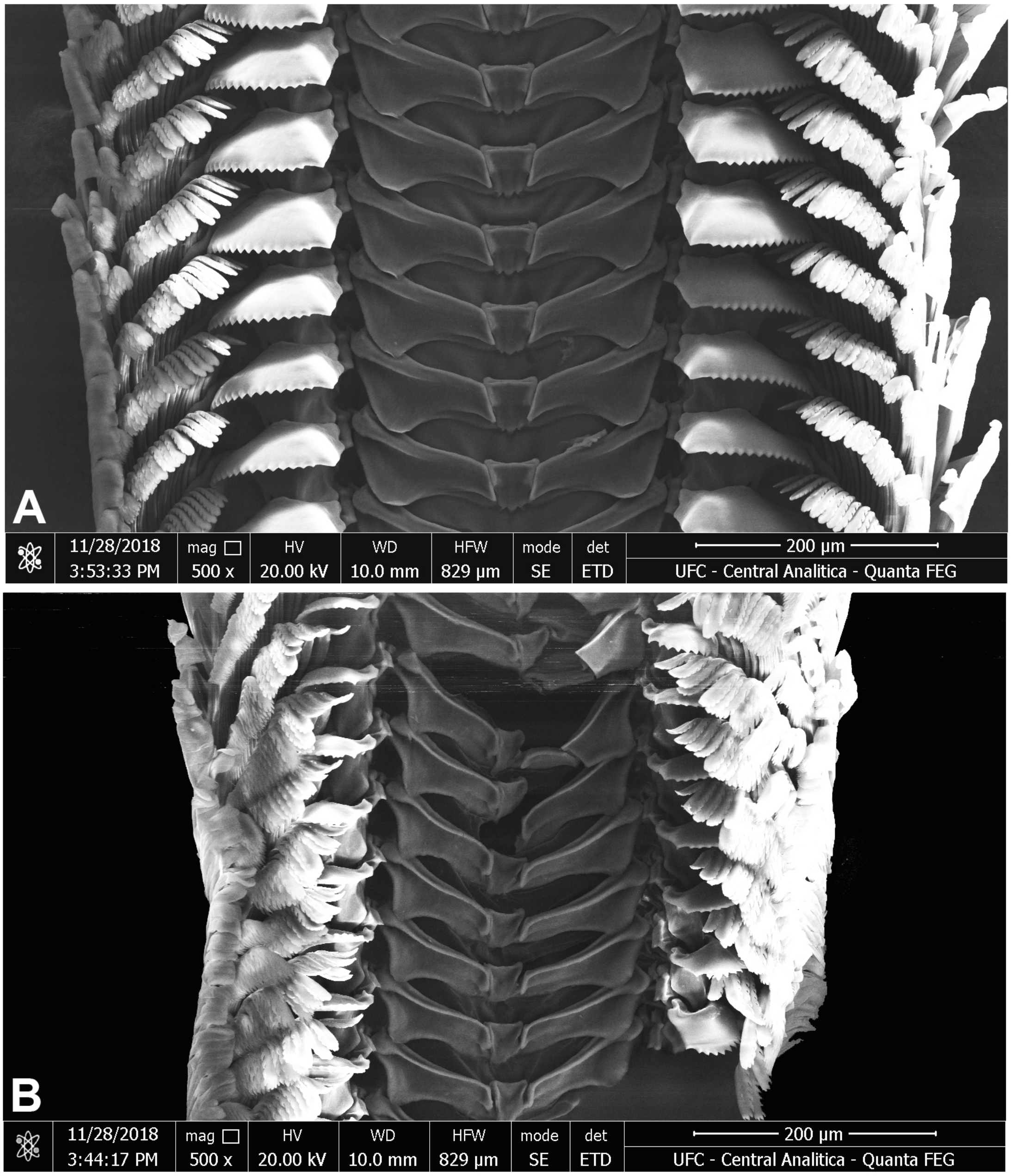

Supplement: Supplementary material 2 [file zookeys-904-117-s002.pdf]
